# Injectable Bioactive Calcium–Magnesium Phosphate Cements Enable Balanced Material Resorption and New Bone Formation

**DOI:** 10.3390/ijms27219974

**Published:** 2026-11-06

**Authors:** Natalia S. Sergeeva, Margarita A. Goldberg, Irina K. Sviridova, Polina A. Krokhicheva, Suraya A. Akhmedova, Valentina A. Kirsanova, Pavel A. Karalkin, Sergey A. Rodionov, Alexandra E. Maltseva, Dinara R. Khayrutdinova, Olga S. Antonova, Aleksander V. Leonov, Ivan V. Mikheev, Vladimir S. Komlev, Andrey D. Kaprin

**Affiliations:** 1 P.A. Herzen Moscow Research Oncology Institute, Branch of Federal State Budgetary Institution “National Medical Research Radiological Centre,” The Ministry of Health of the Russian Federation, 2nd Botkinsky Pass. 3, 125284 Moscow, Russiakirik-57@mail.ru (V.A.K.);; 2 Baikov Institute of Metallurgy and Materials Science, Russian Academy of Sciences, 119334 Moscow, Russiakomlev@mail.ru (V.S.K.); 3 Institute of Cluster Oncology, Sechenov First Moscow State Medical University, Trubetskaya 8, Build 2, 119991 Moscow, Russia; 4 Federal State Budgetary Institution “N.N. Priorov National Medical Research Center of Traumatology and Orthopedics”, The Ministry of Health of the Russian Federation, Priorova Str. 10, 127299 Moscow, Russia; 5 Faculty of Chemistry, Moscow State University, 119991 Moscow, Russiamikheev.ivan@gmail.com (I.V.M.); 6 FSBI National Medical Research Radiological Centre, The Ministry of Health of the Russian Federation, Koroleva Street, 4, 249036 Obninsk, Russia; 7 Department of Oncology and Radiology, Medical Institute, Peoples’ Friendship University of Russia named after Patrice Lumumba, Miklukho-Maklay Str, 6, 117198 Moscow, Russia

**Keywords:** calcium–magnesium phosphate bone cement, injectability, physicochemical properties, bioactivity, cytocompatibility, gene expression, in vivo new bone formation

## Abstract

Injectable calcium–magnesium phosphate cements (CMPC) represent promising materials for bone defect reconstruction due to their bioresorbability, osteoconductivity, and potential to release biologically active Mg^2+^ ions. In this study, injectable CMPCs based on powders containing 20 and 60 mol.% Mg^2+^ were developed using a sodium phosphate solution containing carboxymethylcellulose (CMC) as the cement liquid. The effects of Mg^2+^ content and CMC incorporation on the physicochemical, handling, in vitro, and in vivo properties of the cements were systematically evaluated. CMC acted primarily as a rheology modifier and structuring agent, improving paste cohesion and injectability while maintaining compressive strength suitable for bone substitution. The developed cements demonstrated controlled degradation accompanied by sustained Mg^2+^ release and showed no cytotoxic effects toward MG-63 cells. Biological studies demonstrated favorable cellular responses and osteogenic potential of the developed materials. Importantly, the cement containing the higher Mg^2+^ concentration and CMC demonstrated the most favorable bone defect regeneration outcomes among the investigated formulations, as evidenced by the restoration of mineralized structures observed by micro-CT and the histological identification of newly formed bone tissue. These findings demonstrate that combining increased Mg^2+^ content with CMC-mediated control of cement handling properties is a promising strategy for developing injectable bioactive bone substitutes.

## 1. Introduction

In Russia, the incidence of primary malignant bone tumors is 1.03 cases per 100,000 population, which is consistent with incidence data in other countries [1]. Restoration of postoperative bone defects in onco-orthopedics and maxillofacial neoplasms remains a common clinical problem and is a truly complex task. After surgical tumor removal, extensive defects often form, which are repaired using various reconstructive plastic surgery approaches and modern biomaterials [2]. Currently, autologous bone grafts and allografts are the preferred options [3,4,5]. The “gold standard” for complex defects is the transplantation of the patient’s own tissue. Autotransplantation is widely used for extensive bone defects such as diaphyseal defects of long bones, resection arthrodesis, tumor damage to the distal radial bones, etc. The lack of immunogenicity risk of infection of such biological material, and the high graft engraftment rate are clear advantages of using autologous material. However, the need for a second surgical site, the limited amount of bone graft, additional patient suffering, and an increased hospital stay are its limitations [3]. The use of allografts is associated with the risk of infection transmission, as well as a decrease in the osteoconductivity of the material due to the technology of its preoperative processing [6].

When the use of auto- or allografts is impossible, various synthetic biomaterials are used, including calcium phosphate ceramics [7,8], of synthetic polymer materials [9], composite materials [10,11,12,13] and bone cements [14,15,16].

The use of bone cements in reconstructive and plastic surgery is not limited to the maxillofacial region and the musculoskeletal system [17]. Clinicians currently see great potential for their application in kyphoplasty and vertebroplasty. These surgical interventions are modern minimally invasive procedures introduced for the treatment of osteoporotic vertebral compression fractures, as well as defects caused by oncological diseases [18,19]. The goal of vertebroplasty is to stabilize the fractured vertebral body, while the goal of kyphoplasty is not only to stabilize the fractured vertebral body but also to restore its height to the maximum possible pre-injury level [20]. Both procedures involve the injection of a hardening injectable bone cement (IBC) directly into the vertebral defect, which supports the process of osteogenesis [21,22]. In addition to providing a supporting function, the introduction of bone cement into the vertebral body leads to a significant reduction in the severity of pain syndrome [23]. This feature of the intervention has made it possible to use the introduction of composite materials in osteoporosis and metastases in vertebral bodies. In recent years, vertebroplasty and kyphoplasty have been used in primary and metastatic tumors of the spinal bodies, “critical” spinal osteoporosis, and traumatic injuries of vertebral bodies [24]. Injectable bone cements also find application in procedures for bone tissue augmentation (for example, in orthopedic and maxillofacial surgeries) and bone reconstruction (for example, in filling a bone cyst) [25,26].

The list of requirements for IBCs, according to specialists [27,28], is extensive. However, by general consensus, the most important of these properties are: ease of injection, high radiopacity, viscosity of the hardening cement paste, the rate of bioresorption, which should be coordinated with the rate of de novo bone tissue formation, and mechanical properties comparable to those of healthy bone tissue.

Bone cements can be divided according to their chemical composition into: acrylic bone cements (ABCs) based on polymers, such as polymethylmethacrylate (PMMA), calcium phosphate cements (CPCs), calcium sulfate cements (CSCs), and composite bone cements (CBCs).

ABCs are successfully used in the field of orthopedics [29,30]. PMMA-based bone cements are widely used in modern orthopedic surgeries and degenerative spinal diseases. PMMA-based bone cement is usually represented by two components—a liquid monomer and a powdered polymer [31,32]. PMMA-based bone cements are characterized by rapid polymerization and injectability, which gives bone cement numerous advantages over other materials, especially for use in minimally invasive orthopedic surgery. However, PMMA cements exhibit insufficient biological activity, are unable to fully integrate into bone tissue, and also possess an undesirable thermal effect: the polymerization of the acrylic monomer (which determines the setting process of the cement mass) is accompanied by a strong exothermic effect, which can lead to damage followed by necrosis of soft tissues (in some cases the temperature reaches 80 °C) [33,34]. Also, the contact of the monomer with surrounding tissues leads to the development of dermatitis, and monomer vapors can affect the contact lenses of the operating surgeon [35]. The harmful effect of volatile monomer vapors on the respiratory tract has been previously reported [36].

CPCs have found wide application for the restoration of bone tissue defects [37,38]. The choice of calcium phosphates for use in orthopedics and traumatology is due to their unique properties: these materials are biocompatible and bioresorbable (to one degree or another, depending on the phase composition and microstructure), and they provide a source of calcium and could be substituted by native bone tissue [39,40,41].

Recently, in biomaterials science, close attention has been paid to magnesium-based materials. This is due to the ability of magnesium to fully resorb in the human body with the release of Mg^2+^ cations, which positively influence osteoinductivity in the process of osteogenesis [42], increasing the transformation of nonspecific connective cells, the so-called inducible osteoprogenitor cells, under the action of bone morphogenetic protein (BMP) [43,44]. Bone marrow-derived mesenchymal stem cells (BM-MSCs) are of paramount importance for bone tissue regeneration. In response to specific stimuli, such as BMPs, they undergo osteogenic differentiation. Magnesium, its transporter protein MagT, and the transient receptor potential cation channel TRPM7 play a crucial role in this process [45,46].

A study investigating the signaling pathways associated with the anti-inflammatory and immunomodulatory properties of magnesium ions has identified the TRPM7-PI3K-AKT1 axis, the activation of which leads to reduced levels of IL-6 and TNF [47]. The immunomodulatory properties of Mg^2+^ during bone tissue regeneration are mediated by the polarization of macrophages from the M1 to the M2 phenotype and the upregulation of CD206 and the anti-inflammatory cytokine IL-10, which leads to increased osteoblast activity while suppressing osteoclasts [48,49].

Mg2+ also promotes the differentiation of terminal capillary cells during vessel formation through crosstalk between the VEGFA-VEGFR2 and Notch1 signaling pathways, thereby enhancing angiogenesis in the zone of bone regeneration. Mg^2+^ [46,50].

Thus, the magnesium within the implants enhances bone tissue regeneration during the process of bioresorption by activating signaling pathways in mesenchymal stromal cells and modulating the immune response.

It is expected that materials incorporating Ca, Mg, and P will possess good biocompatibility, the ability to fully resorb under body conditions, and exhibit bioactivity with respect to the formation of a healthy bone matrix during reparative osteogenesis, which are the main requirements for materials used for bone defect replacement [51,52,53].

To date, the bone cement OsteoCrete™ from Bone Solutions, Inc. (BSI) (Colleyville, TX, USA) has been registered with the FDA (Pure Food and Drug Administration, USA) and is clinically used for bone defect replacement [54]. OsteoCrete bone cement consists of 87% potassium magnesium phosphate cement (MPC) (that is, magnesium oxide and potassium phosphate), and also contains 13% other ingredients that are a trade secret. This composition is characterized by high adhesive properties and is used for tendon-to-bone fixation, bone flap fixation, and screw fixation.

This assumption rests on the greater solubility of biocompatible magnesium phosphate minerals under in vivo conditions, which accelerates resorption and bone tissue remodeling through the release of magnesium ions [43]. A number of in vivo animal studies, including those on tibial defects in sheep and pigs, have confirmed this behavior [55,56,57].

By incorporating both calcium and magnesium phosphates, CMPCs demonstrated improved functional and exploitation properties, including high strength and optimal setting time, bioresorption kinetics, biocompatibility and osteoconductivity [58]. Further development of the scientific foundations of such CMPCs is associated with the creation of injectable CMPCs. The approach linked with introduction of polymer is one of the most promising [59,60], it was shown that several materials contribute to increasing the injectability of cements, including sodium CMC, hydroxypropylmethylcellulose (HPMC), chitosan, glycerol, and salts of alginic acid (most often sodium alginate). HPMC and CMC are derivatives of cellulose, one of the most common and affordable polysaccharides. CMC is non-toxic and biocompatible, does not elicit an immune response, could be sterilized without significant degradation [61,62]; therefore, it is used in surgery.

In a clinical situation, the surgeon’s ability to correctly mix the cement powder with the cement liquid and then place the cement paste into the defect within the prescribed time is a decisive factor for achieving optimal surgical results. Thus, it would be desirable to have a cement paste premix that is stable in packaging and hardens only after placement in the bone defect. Such a paste can be created by combining CPC powders with a non-aqueous but water-miscible cement liquid, since such cements harden only during hydration. In work [63], it was shown that a cement paste based on tetracalcium phosphate [Ca_4_(PO_4_)_2_O, TeTCP] and anhydrous dicalcium phosphate is in a stable state in a syringe and hardens only after it is delivered into the root canal, where it interacts with water from the surrounding tissues. The cement liquid was prepared based on a solution of the sodium salt Na_2_HPO_4_ with the addition of glycerol with different ratios of HPMC in the composition; the setting time of such cements was from 30 min and increased to 100 min as the polysaccharide was added to the cement liquid composition; however, all the obtained cement materials possessed insufficient mechanical characteristics (compressive strength was 2.6–6.5 MPa).

It is expected that a more viscous IBC will perform better than a low-viscosity IBC [64]. There are three mechanisms [65,66] through which additives improve cement cohesion: (1) increasing the viscosity of the liquid phase of the cement; (2) adsorption on cement grains and enhancement of attraction between particles; (3) addition of ultrafine particles with a large surface area to the cement paste.

The use of viscosity-increasing additives such as polyvinylpyrrolidone, hydroxypropylmethylcellulose, CMC, and starch was effective in increasing the cohesion and stability of cement materials [63,67]. These additives increase the water-retaining capacity of the cement and reduce the risk of heterogeneous separation. Water-soluble polysaccharides are used as viscosity-increasing additives to improve the cohesion and stability of cements [68].

Research in the field of creating injectable CMPCs is also based mainly on optimizing the powder/liquid ratio or on introducing a polymer into the cement composition. As cement powders, a mixture of CPC and MPC is used, where the MPC powder is based, for example, on MgO [69] or MgO and NH_4_H_2_PO_4_, which during the reaction form the ammonium-containing phase struvite NH_4_MgPO_4_·6H_2_O, which is characterized by undesirable release of ammonium during bioresorption in the body [70]. As polymers for CMPCs based on mixtures of CMC and MPC, consisting of tetracalcium phosphate and dicalcium phosphate dihydrate, as well as MgO and Ca(H_2_PO_4_)_2_, white dextrin [69] and xanthan gum have been proposed. In cements containing white dextrin, improved resistance to washout and injectability were shown compared with CPC, and for cements containing xanthan gum, in vivo tests were also conducted, and osteoconductivity was established in a rabbit tibia model [71]. Studies of in situ porosity formation were not conducted in these works.

Thus, the aforementioned publications describe, to varying degrees, the physicochemical and biological properties of CMPCs. Similar studies of injectable bone cements containing magnesium ions and CMC as a setting liquid are still isolated.

The aim of this work is to systematically investigate CMPCs with varying magnesium ion content, using CMC as the cement liquid component, and to evaluate the relationship between the physicochemical properties of the resulting materials and their in vitro and in vivo bioactivity.

## 2. Results

### 2.1. Characterization of Cement Powders

The chemical composition, phase composition, and particle size distribution of the initial cement powders were similar to those developed and presented earlier [72]. Briefly, the powder containing 20 mol.% Mg^2+^ consisted predominantly of the Mg-substituted whitlockite phase (Mg-Wt, Ca_2_._589_Mg_0_._411_(PO_4_)_2_, ~85 wt.%), whereas stanfieldite (St, Ca_3_Mg_3_(PO_4_)_4_) was the main phase in the powder containing 60 mol.% Mg^2+^. MgO was detected as a minor phase (5–10 wt.%) in both compositions. Laser diffraction analyses showed that the powders contained no particles larger than 100 μm, with characteristic particle sizes of approximately 20 μm and 50 μm for the 20 and 60 mol.% Mg^2+^ compositions, respectively. Further details on the powder synthesis and characterization can be found in our previous work [72,73].

### 2.2. Characterization of Cement Liquids

Cement liquids with different concentrations of CMC polymer (0.25, 0.5, 0.75, and 1.0 wt.%) were investigated. Solutions containing CMC were prepared with a higher polymer concentration and were characterized by a uniform distribution of components throughout the volume. Mixing the aqueous CMC solution with the cement liquid based on the NaH_2_PO_4_ solution did not result in gelation or turbidity, suggesting that no chemical crosslinking of the polymer occurs. The intermolecular interactions are confined to ion-mediated phenomena, namely the redistribution of sodium counterions and electrostatic modulation of the polymer chain conformation (further confirmed by FTIR, Section 2.3). Viscosity measurements (Table 1) showed that the resulting solutions are non-Newtonian fluids. As the CMC content increased, the viscosity of the solution rose, reaching 235.5 ± 0.5 mPa s at a rotation speed of 25 rpm upon the introduction of 1.0% CMC. At a rotation speed of 100 rpm, viscosity growth was observed in the concentration range of 0–0.5% and was changed insignificantly at 0.75 wt.% CMC. Further increases in CMC content led to a decrease in viscosity, reaching a minimum value of 15.7 mPa s at a concentration of 1% CMC. This behavior indicates the formation of non-Newtonian fluids upon the introduction of high CMC concentrations, characterized by a nonlinear dependence of viscosity on rotation speed and biopolymer concentration. Thus, the introduction of 0.75–1.0% CMC gives rise to a system characterized by structural viscosity. Surface tension and pH of the liquids are listed in Table 1. The introduction of CMC at a minimum concentration of 0.25% in sodium phosphate solution led to a decrease in surface tension. At the same time, as the CMC content increased, the surface tension rose and reached a value of 77.9 mN/m.

The initial acidic pH of the polymer-free liquid (~3.4) is determined by the presence of NaH_2_PO_4_, which in aqueous solution establishes an equilibrium between H_2_PO_4_^−^, HPO_4_^2−^, and H^+^ ions, maintaining a weakly acidic environment. The introduction of CMC, which contains Na^+^ and carboxyl groups (–COOH) with a pKa of approximately 4.0–4.5, introduces an additional acid–base equilibrium into the system. As the polymer dissolves, the carboxyl groups partially dissociate, releasing H^+^ simultaneously release the alkali Na^+^ ions from CMC salt.

However, the presence of the phosphate ions can consume some of these protons, shifting the equilibrium and contributing to the observed pH increase. Importantly, the final pH of ~4.0 is close to the pKa of both the carboxyl groups of CMC and the phosphate buffer system, suggesting that the system reaches a buffered state where both components coexist in their partially protonated/deprotonated forms. It was found that the addition of CMC contributes to an increase in the pH level of the cement liquid; solutions containing 0.5% or more CMC exhibited pH values in the range of 3.9–4.1 (Table 2).

It was also established that an increase in polymer content has a minor effect on the wettability of glass and Teflon surfaces by the cement liquids, slightly reducing the contact angle between the liquids and both types of surfaces (Table 2). Accordingly, a CMC concentration of 0.75% was selected for subsequent investigations of the cement materials as it ensures a high additive content without causing a dramatic increase in viscosity and sufficient contact angles for both surfaces.

### 2.3. Characterization of Cement Materials

Cement materials with the following names were obtained: Cem1 (20%Mg+A), Cem2 (60%Mg+A), Cem3 (20%Mg+A+CMC) and Cem4 (60%Mg+A+CMC), respectively.

**Figure 1:**
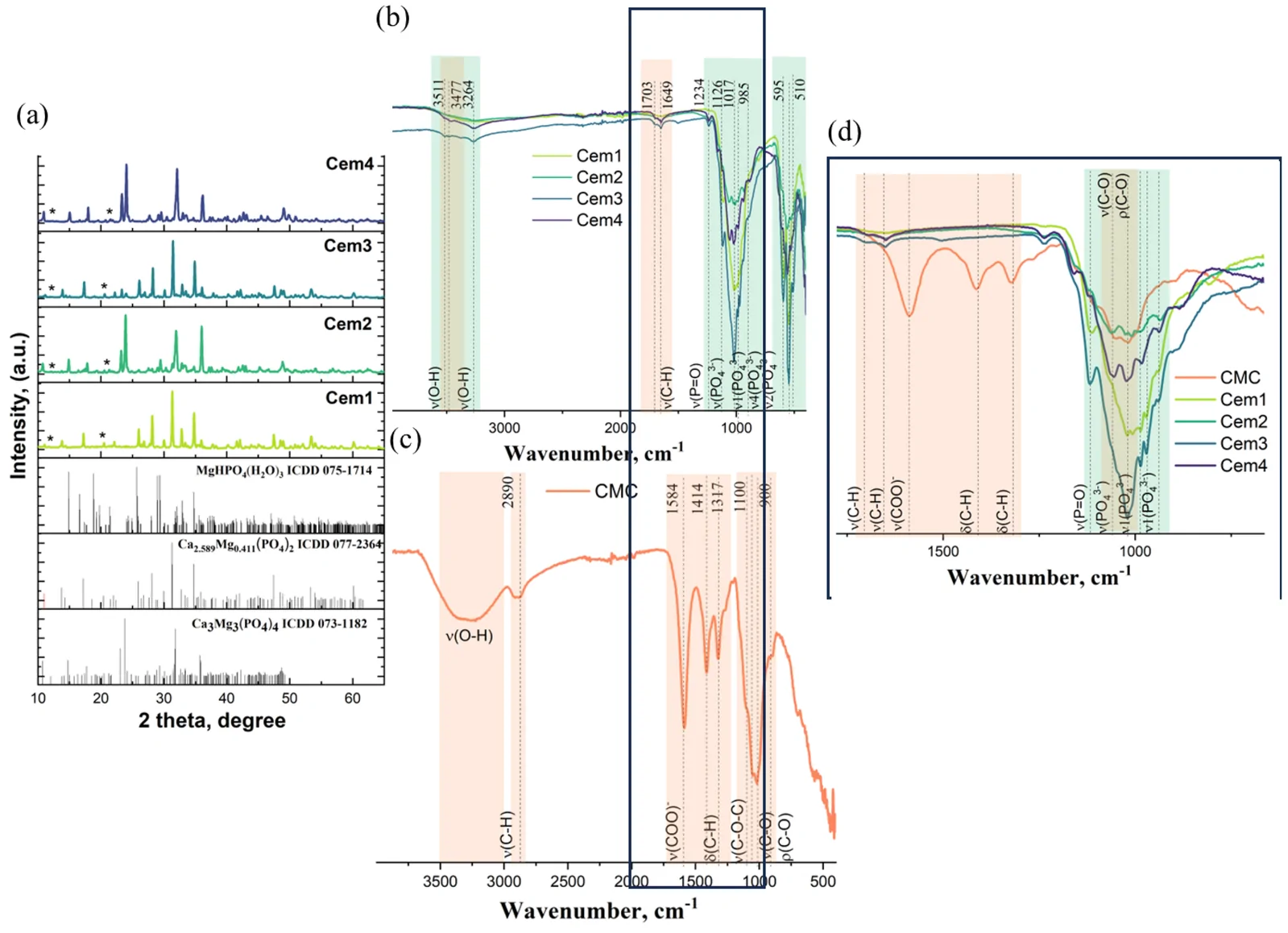
The cement phase composition according to XRD: *— brushite phase (**a**); FTIR investigation of CMC polymer (**b**); cements Cem1-Cem4 (**c**); more detailed FTIR results for the investigated system (**d**).

Investigation of the phase composition of cement materials obtained from powders containing 20 and 60 mol.% Mg without CMC (Cem 1 and Cem 2) and with the addition of CMC in the amount of 0.75 wt.% (Cem 3 and Cem4) revealed the formation of new setting phases: newberyite (MgHPO_4_·3H_2_O), and brushite (CaHPO_4_·2H_2_O). Newberyite is a phase of orthorhombic symmetry with theoretical lattice parameters a = 10.2048(4) Å, b = 10.6849(3) Å, c = 10.0135(7) Å (Figure 1a). The formation of newberyite as the initial setting phase is consistent with the literature on MPC, where the reaction between MgO and phosphate salts in aqueous media proceeds through the formation of magnesium hydrogen phosphate hydrates [44].

In the presence of a Mg-whitlockite or stanfieldite phase in the initial cement powder, the formation of newberyite occurs additionally through the acid–base reaction with the NaH_2_PO_4_-based liquid phase. The reaction can be represented as follows Equations (1) and (2):

Ca_x_Mg_y_(PO_4_)_3_ + (2x + 3y + 3)NaH_2_PO_4_ + (4x + 3y + 3)H_2_O → xCaHPO_4_ × 2H_2_O + yMgHPO_4_ × 3H_2_O + (2x + 3y + 3)Na_2_HPO_4_ (1)

Mg_3_Ca_3_(PO_4_)_4_ + 7NaH_2_PO_4_ + 13H_2_O → 2CaHPO_4_ × 2H_2_O + 3MgHPO_4_ × 3H_2_O + 7Na_2_HPO_4_ (2)

The dominant phase in cement materials Cem 2 and Cem4 was stanfieldite, present in an amount up to 81 wt.%. For cement materials Cem1 and Cem3, the main phase was Mg-Whitlockite in the amount of ~31 wt.%. Also, the minor content of the brushite (CaHPO_4_·2H_2_O) phase was detected. This phase crystallized according to Equations (1) and (2) or precipitated from dissolved calcium-containing phases due to acid–base reaction between the cement powder and the cement liquid:

Ca^2+^ +H_2_PO_4_^−^ +H_2_O → CaHPO_4_ × 2H_2_O + H^+^ (3)

The precipitation of brushite occurs as a result of the reaction of calcium ions released from the powder with dihydrogen phosphate ions present in the liquid phase, accompanied by the release of protons. This reaction is typical for brushite-forming CPC [74,75,76] and is responsible for the acidic pH environment during the early stages of setting. Although brushite is more soluble, it is a metastable phase and can transform to an apatite phase in vivo [77,78].

The FTIR investigation of CMC and cement materials was assessed (Figure 1b–d). Sodium phosphate was employed as the PO_4_^3-^ source to facilitate phosphate phase formation. Notably, no bands indicative of crystalline sodium phosphate species (for instance, in the 1200–1150 cm^−1^ range) were observed in the FTIR spectra. This finding implies that the dominant phosphate phases are those of calcium and magnesium.

The FTIR spectrum of neat CMC shows the typical absorption features of the polymeric matrix (Figure 1b) [79,80,81]. A broad and intense band centered in the 3500–3000 cm^−1^ range is ascribed to O-H stretching vibrations, reflecting the presence of hydrogen-bonded hydroxyl groups. The peaks observed at 2920 and 2850 cm^−1^ originate from the asymmetric and symmetric C-H stretching modes of the methylene units. Notably, two distinctive bands located at 1600–1590 and 1420–1410 cm^−1^, attributable to the asymmetric and symmetric stretching of the carboxylate moiety (-COO^−^), serve as clear evidence for the preservation of carboxymethyl groups within the polymer structure. Additionally, the bands in the 1160–900 cm^−1^ region, arising from C-O-C and C-O stretching vibrations, confirm the intact glucopyranose backbone of CMC. The FTIR spectra of the cement samples Cem1–Cem4 show intense absorption bands typical of the phosphate anion (PO_4_^3−^) (Figure 1c). In the high-wavenumber region associated with stretching modes (1200–900 cm^−1^), a broad multiplet centered around 1120–1000 cm^−1^ is evident, which corresponds to the asymmetric stretching vibrations (ν_3_) of the PO_4_^3−^ group. A distinct band at 960–950 cm^−1^ is attributed to its symmetric stretching mode (ν_1_). In the low-wavenumber region (650–500 cm^−1^), the deformation vibrations of the phosphate tetrahedron are manifested as characteristic bands at 605–600 and 570–560 cm^−1^ (ν_4_). Compared to undoped CP [82] the CMP samples exhibit notable broadening and reduced intensity of the phosphate bands, especially in the 1100–1000 and 600–550 cm^−1^ ranges. These spectral changes point to a decreased crystallinity of the phosphate phase and, presumably, to partial substitution of Ca^2+^ by Mg^2+^ within the crystal lattice. Collectively, the observed bands provide unambiguous evidence for the formation of calcium and magnesium phosphate phases in the cement samples. Moreover, a band at 982 cm^−1^, assigned to the phosphate anion in the brushite structure, corroborates the presence of this phase in the samples; more pronounced peaks were observed for Cem1 and Cem2.

The FTIR spectra of CMC-containing cement samples Cem3-Cem4 reveal several spectral changes pointing to interactions between the components. Most notably, the O–H band (3600–3000 cm^−1^) undergoes significant broadening and a reduction in intensity, indicating the participation of CMC hydroxyl groups in hydrogen bonding with phosphate ions at 3477 cm^−1^. In addition, the carboxylate bands at 2890, 1600–1590 [83] and 1420–1410 cm^−1^ display minor shifts and altered profiles, which are most reasonably ascribed to electrostatic interactions between the carboxylate moieties of CMC and Ca^2+^/Mg^2+^ cations at the surface of growing phosphate particles (Figure 1d). At the same time, the phosphate bands—especially ν_3_ and ν_4_—become broader, a feature typical of amorphous or poorly crystalline phosphate phases. These observations collectively indicate that CMC acts as a polymeric matrix that preserves the phosphate phase in an amorphous form and hinders its crystallization.

**Figure 2:**
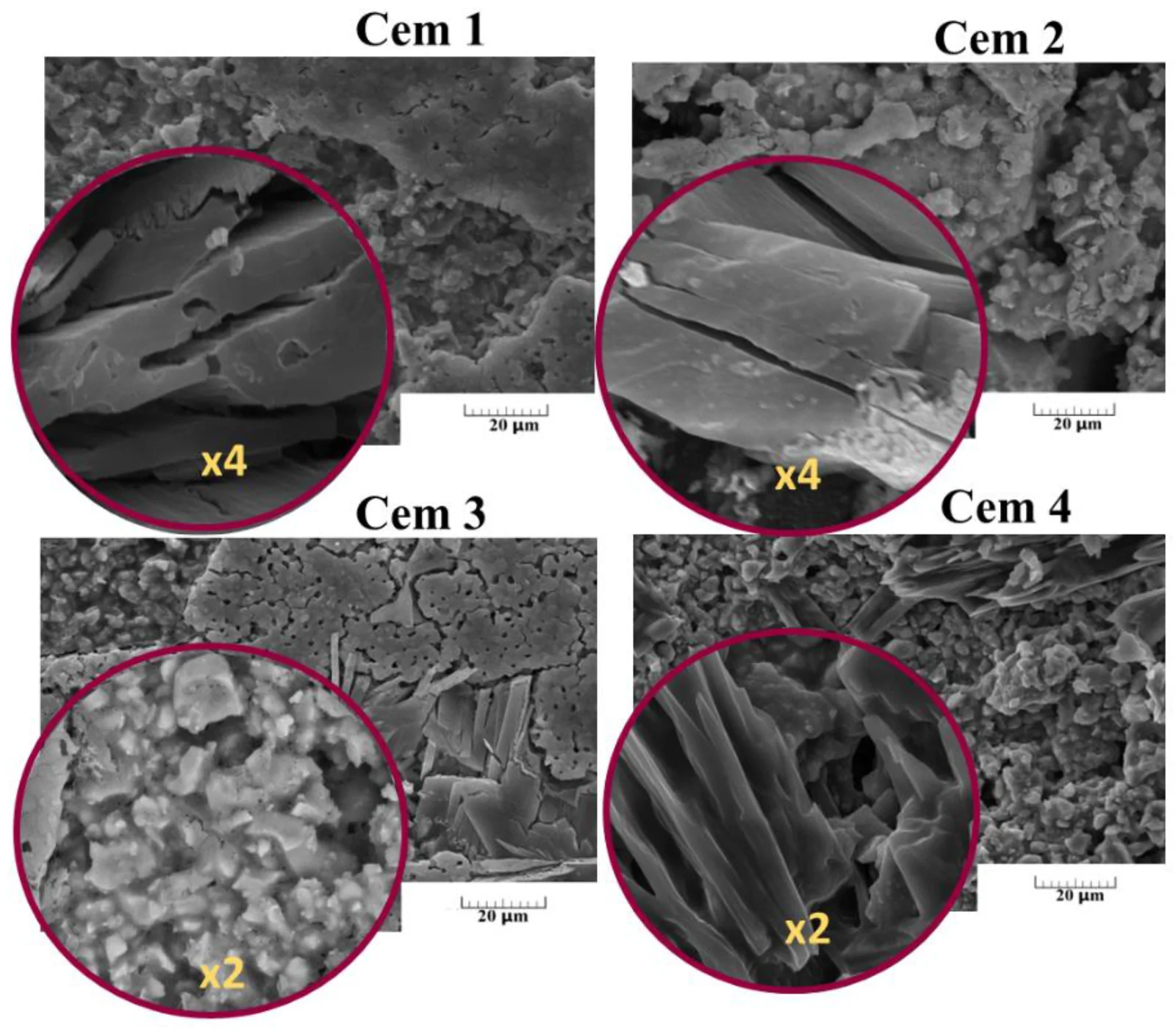
Microstructure on the surface of the cement samples according to SEM investigation in the secondary electron’s mode.

The introduction of the polymer into the cement liquid led to the appearance of amorphous regions on the micrographs of the cement sample surfaces (Figure 2). Zones of interaction between the cement powder and the liquid were visible, along with the formation of prismatic crystals, as well as finer, more rounded crystallites for all cement compositions. The plate-like crystals observed in Cem1 and Cem2 could be attributed to the brushite phase, which typically exhibits a triangular or trapezoidal, tabular morphology in the monoclinic system. This morphological assignment is in good agreement with the FTIR data, where the PO_4_^3−^ stretching vibrations at 982 cm^−1^ corresponding to acidic calcium phosphates were observed for the Cem1 and Cem2 compositions. Meanwhile, the finer, rounded crystallites in Cem3 could correspond to newberyite phase, which crystallizes in the orthorhombic system with a pseudohexagonal or rosette-like habit. In summary, it could be concluded that CMC inhibited anisotropic growth of crystals. This is consistent with the XRD data, which showed lower peak intensities for CMC-containing samples, reflecting a higher proportion of amorphous material.

**Figure 3:**
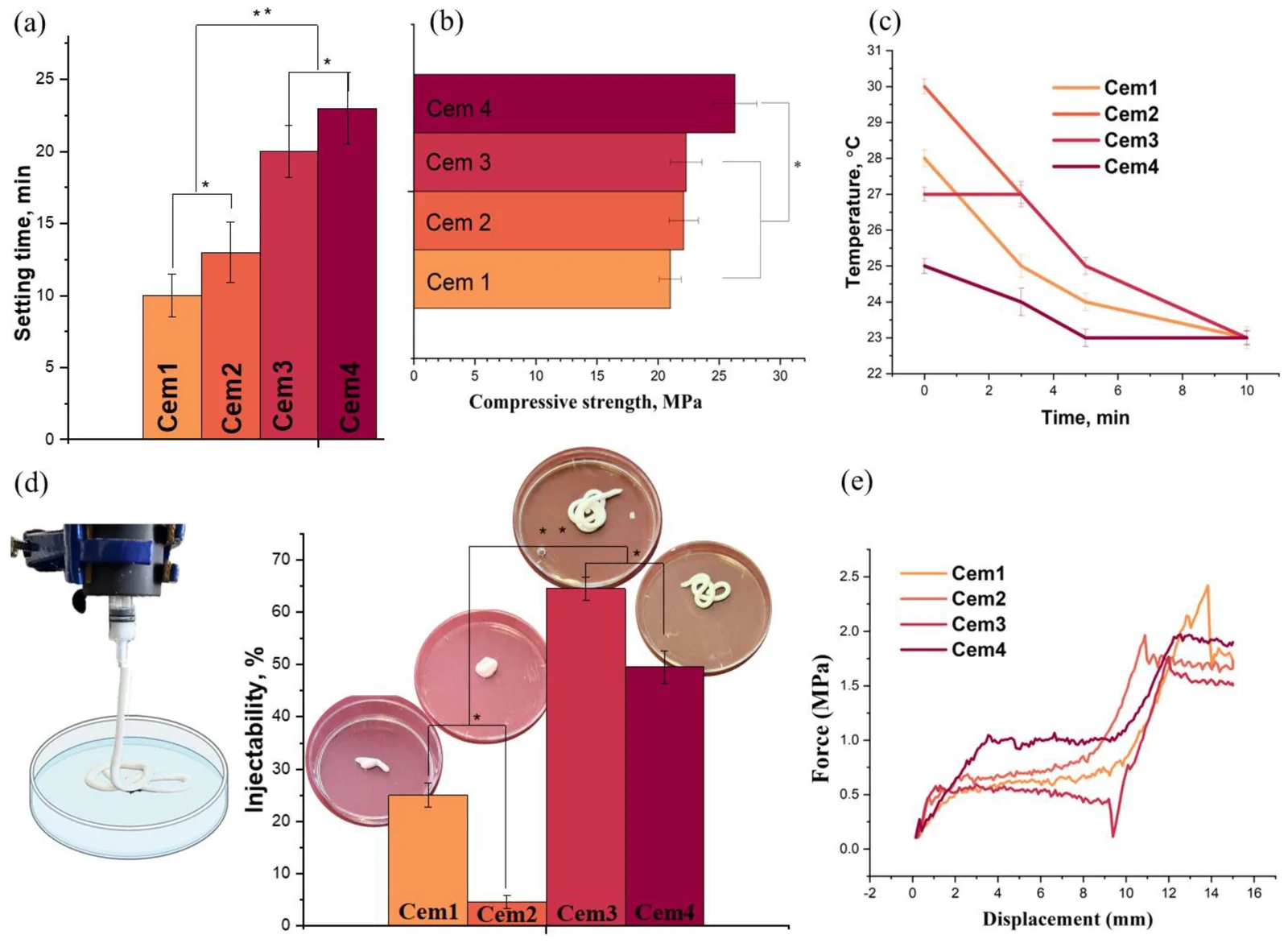
The influence of CMC on the cement properties: the setting time (**a**), the compressive strength (**b**), the setting temperature (**c**), the digital photo of the cement filament after the injection and injectability test, injectability of the cement samples, where * is a significant difference between the cements without CMC and with CMC (*p* < 0.05),** is a significant difference between the cements based on the powders contained 20 and 60 mol.%Mg^2+^, (*p* < 0.05) (**d**), and the influence of the composition on the injection force (**e**).

The influence of polymer incorporation on the properties of cement materials, specifically setting time (Figure 3a), compressive strength (Figure 3b), temperature (Figure 3c), and injectability (Figure 3d,e) was assessed. The cement material based on the powder containing 20 mol.% Mg without CMC (Cem1) exhibited a following properties: setting time in the range of 8–12 min, a compressive strength of approximately 20 MPa, the temperature in the moment of mixing is about 30 °C and becomes 23 °C after 10 min of setting, an injectability of 20%. The addition of CMC led to an approximately twofold increase in setting time, a slight decrease in the initial setting temperature, improvement in mechanical properties, and a noticeable increase in injectability up to 63–65%.

Cement material based on the cement powder containing 60 mol.% Mg without CMC (Cem2), showed a setting time of 13–15 min, a compressive strength of about 23 MPa, and a very low injectability with a value of less than 5%. This behavior is attributed to the larger particle size of the initial powder (D90 = 57.6 µm [72]). The addition of CMC to the Cem2 formulation increased compressive strength to 26 MPa, injectability to 50%, and extended the setting time to more than 20 min. Powders containing 20 mol.% Mg (characterized by the finer particles with D90 of 18.6 µm) have a surface area and a greater number of active sites for polymer adsorption. This means that, at the same CMC concentration, a denser and more continuous polymer layer could be formed on the surface of finer cement powder particles. As a result, the setting retardation effect may be more pronounced for Cem3 [84,85]. Also, it was detected the decreasing in the initial setting temperature to 25 °C, which was measured during the mixing of 60 mol.% Mg cement powder with the cement liquid.

The optimal injectability of cement pastes should approach 100%, corresponding to complete extrusion of the material. A critical issue to avoid is the “filter pressing” effect, which describes the undesirable separation of the liquid and solid phases within the syringe. However, the paste must also exhibit sufficient viscosity to prevent spontaneous leakage upon extrusion. During the research, we found a considerable correlation between the initial particle size of the cement powders and the maximum injection force of the cement paste. The extrusion pressure can be estimated from the force–displacement curves (Figure 3e). The Cem1 samples exhibited the highest value of 2.4 N/mm^2^ at a displacement of 15 mm, reflecting considerable resistance during injection. For Cem2, however, the peak force was reached earlier, at 10 mm of displacement, which indicates that compaction of the cement paste within the syringe had already begun, thereby compromising the injectability of the material. In contrast, the Cem3 and Cem4 formulations required a maximum force of only 1.5 N/mm^2^ and 1.7 N/mm^2^ for 12 mm of displacement, respectively. This reduction suggests that the inclusion of CMC in the liquid phase enhances the wettability of the cement powder, thereby improving injectability while simultaneously lowering the required extrusion pressure. Cement powder with a smaller particles exhibits enhanced reactivity with the cement liquid, resulting in improved wettability and, consequently, better injectability for Cem3.

### 2.4. In Vitro Dissolution Assays

**Figure 4:**
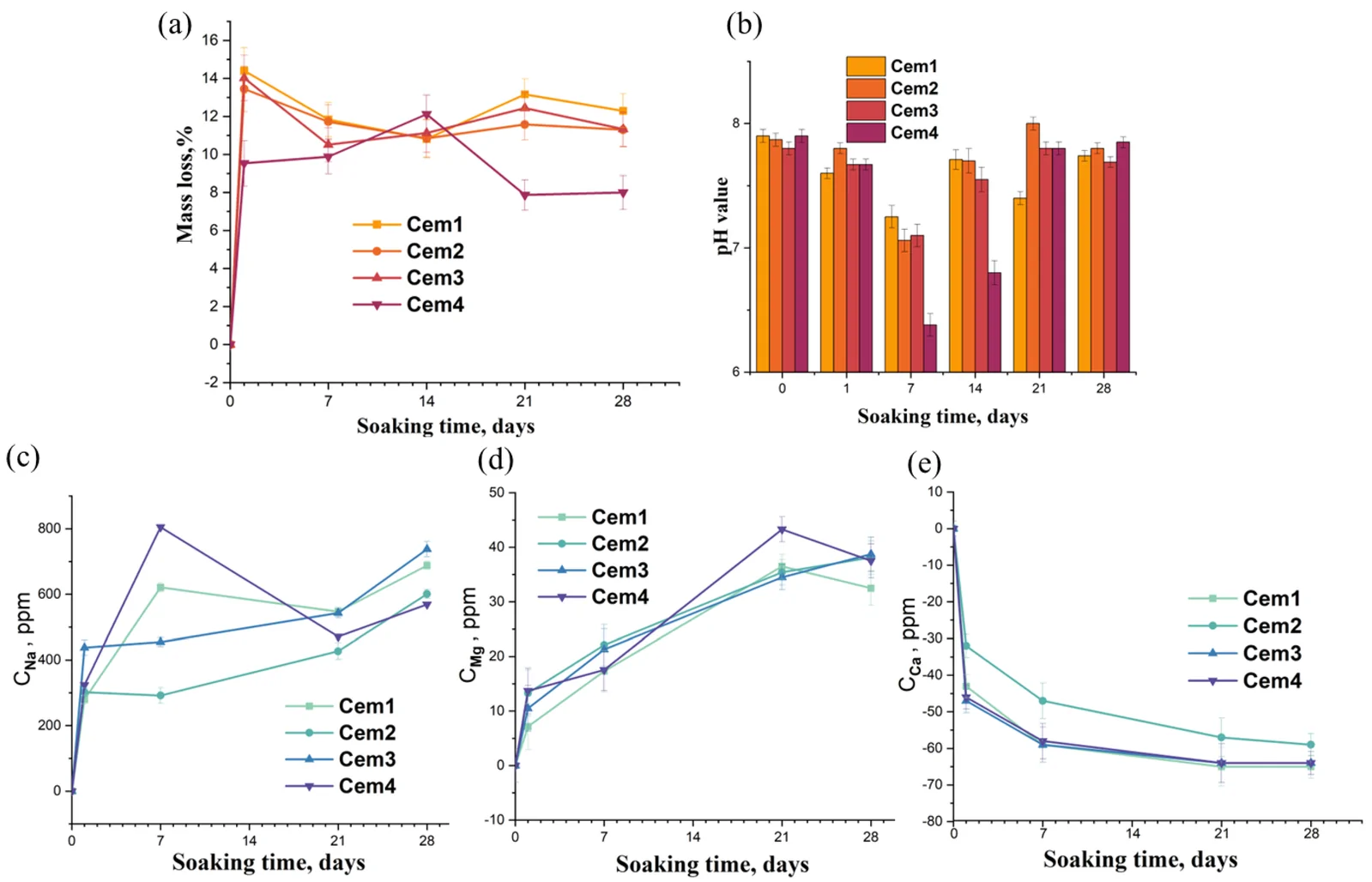
Influence of the CMC on the dissolution kinetics of the cement in the SBF solution: the mass loss (**a**), pH evolution in the SBF (**b**), and the Na^+^ (**c**), Mg^2+^ (**d**), Ca^2+^ (**e**), release into solution according to ICP data.

The degradation kinetic behavior of the cement materials was determined by weight loss (Figure 4a), pH (Figure 4b), and the release concentrations of Ca^2+^, Mg^2+^, and Na^+^ ions (Figure 4c–e), and the profile was plotted as a function of immersion duration. The results of quantitative assessments of the weight loss of cement samples during immersion in SBF solution demonstrate a similar degradation profile regardless of composition, with weight loss in the range of 11–14%. It can be stated that the Cem4 sample exhibited the lowest weight loss, reaching 8% by day 28 of the experiment. The pH changes reflect the dissolution processes of the cement phases, as well as the dissolution of the unreacted cement liquid. By day 7, a slight decrease in pH (from 7.2 to 6.4) was observed, which can be attributed to the dissolution processes of the brushite phase and active release of H^+^ ions into the solution. Release of Na^+^ ions into solution was indicated as a result of leaching of the unreacted cement liquid. This process was most pronounced for the Cem4 samples, where the concentration of Na^+^ in the solution reached 800 ppm at 7 days. Subsequently, at the 7-day point, the pH level began to rise and reached values of 7.8–8.0. Throughout the entire observation period, active release of Mg^2+^ ions into the solution occurred, which is associated with the gradual dissolution of the newberyite phase, with similar dynamics for all compositions. However, a decrease in Ca^2+^ ions in the solution was observed, indicating that calcium ions are actively deposited on the sample surface.

**Figure 5:**
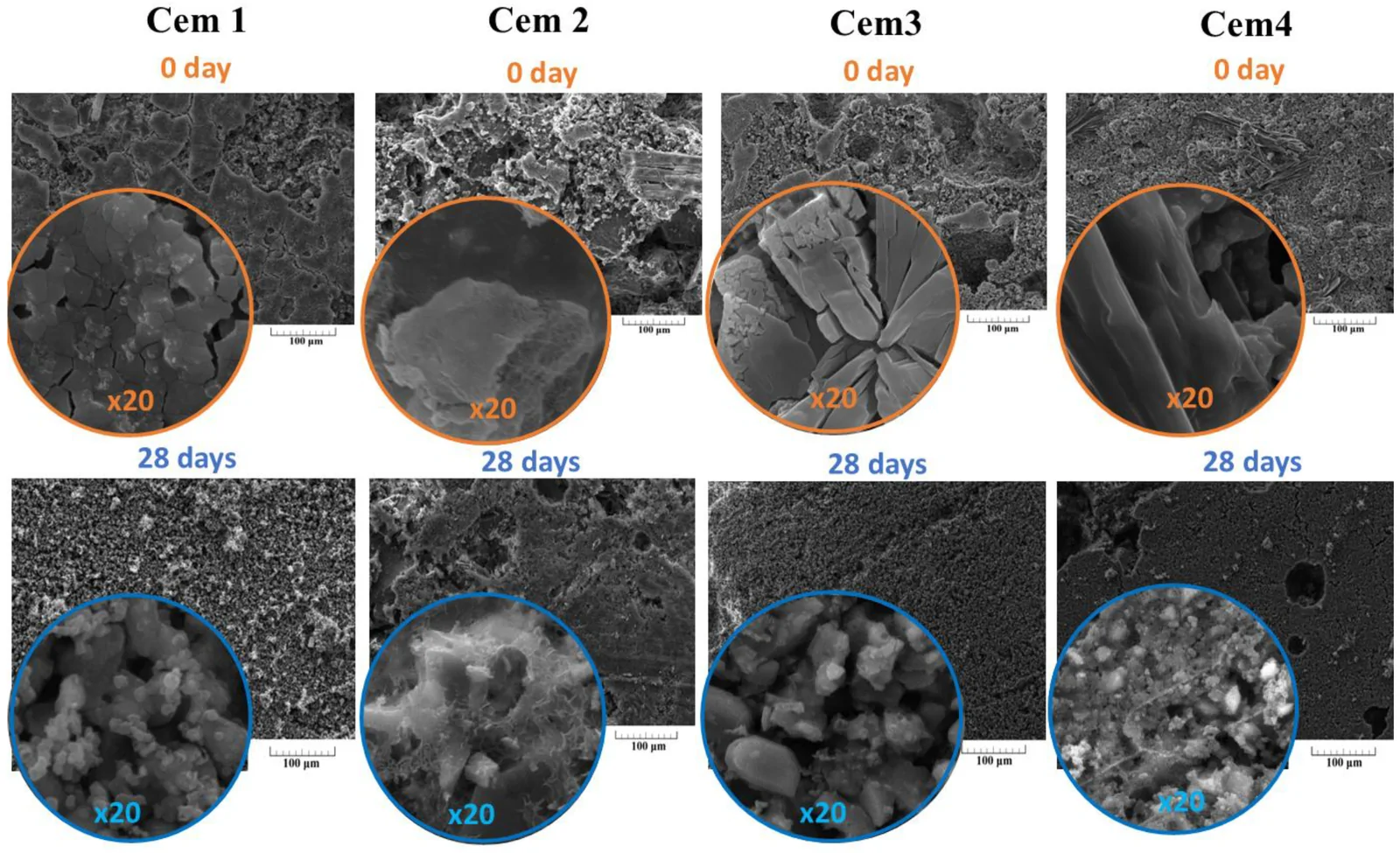
The morphology of the CMPC samples’ surfaces according to SEM during the dissolution study on 0 and 28 days of immersion.

According to SEM data (Figure 5), the surface of cement materials after 28 days of immersion was represented by a fairly porous framework with occasional crystals of a precipitated CP phase, which are more visible for Cem 1 and Cem 2. The precipitation of CP phases on the material surface is a consequence of its interaction with the SBF solution. This process typically begins with the release of Ca^2+^ ions, which, upon reaction with phosphate ions from the SBF, leads to the local supersaturation necessary for the formation of an amorphous calcium phosphate (ACP) precursor phase [86,87]. The ACP subsequently undergoes a phase transformation, often via an octacalcium phosphate intermediate, to form a more stable carbonated apatite. The appearance of precipitated crystals on the surface of Cem 1 and Cem 2 is consistent with the formation of this final apatite phase, which can exhibit plate-like or needle-like morphology. The surface of samples containing the polymer Cem 3 and Cem 4 was denser. This might suggest that the polymer influences the kinetics of the dissolution–precipitation process, potentially leading to a more uniform or compact precipitated layer. It can be concluded that the introduction of the CMC has a minor effect on the overall dissolution process of the cement material, though it appears to alter the characteristics of the surface layer formed.

### 2.5. In Vitro Cytotoxicity of CMPC Samples

In the section on in vitro studies of the biological activity of CMPC samples, information on the Cem1 and Cem2 samples has already been partially published by us [72].

**Figure 6:**
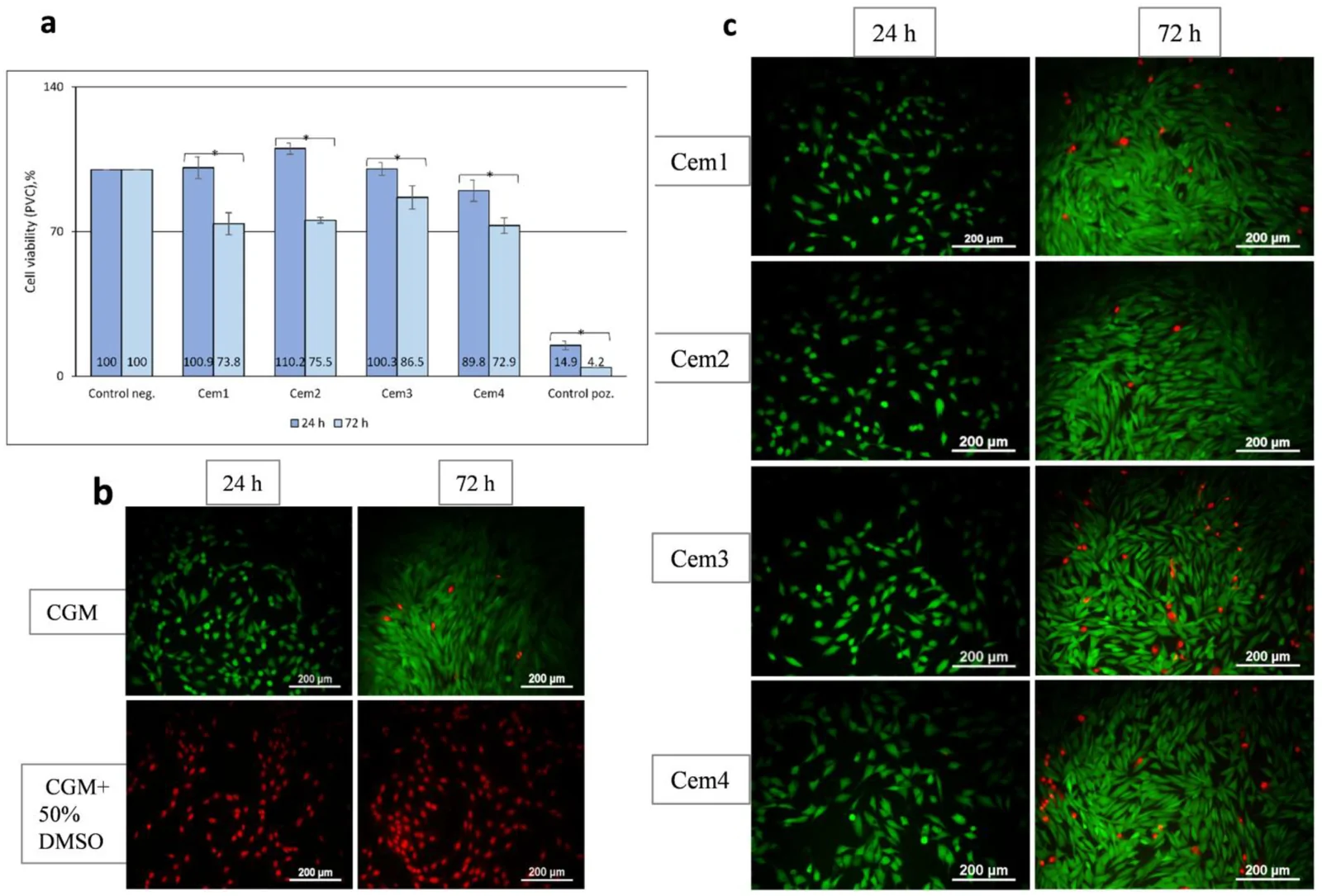
Viability of MG-63 cells after 24 and 72 h of growth in extracts of Cem1, Cem2, Cem3 and Cem4 samples (indirect test): (**a**) population of viable cells (PVC) of MG-63 in relation to control (polystyrene) (MTT test). Negative control—cells on polystyrene in сomplete growth medium (CGM); positive control—cells in 50% dimethyl sulfoxide (DMSO). Error bars represent means ± SD *for n* = 3. *—Differences were identified as significant, which are represented by *p* < 0.05. Border cytotoxicity value (70% of cell viability in CGM) is shown as a horizontal line; (**b**,**c**) fluorescence imaging of the live (green) and dead (red) staining of MG-63 cells using the Live/Dead Kit.

The toxicity of the sealed Cem1-Cem 4 samples was determined in vitro by an indirect assay by culturing MG-63 cells in their extracts for 24 and 72 h [88,89]. It was shown that, according to the MTT test, after 24 h of cell growth under these conditions, the PVC index for the Cem1-Cem3 samples was at the control level (cells on polystyrene in CGM) and amounted to 101.0–103.0%; for Cem 4 (60 wt.%, CMC sealing liquid), it was slightly lower (86.5%). After 72 h of the experiment, the PVC index for all cement samples was statistically significantly lower compared to the previous period and was in the range of 72.9–86.5% (Figure 6a). Since the obtained PVC values for these bone cement samples are above the discrimination level (70%), it can be concluded that they are not toxic for this test culture. The obtained results also coincide with the data of the Live/Dead test (Figure 6b,c): the presented micrographs show that after both 24 h and 72 h of MG-63 cell growth in the presence of extracts of Cem1-Cem4 samples, the population of living cells (green fluorescence) predominates.

### 2.6. In Vitro Study of the Effect of CMPC Samples on the Migratory Capacity of MG-63 Cellsin Scratch Test

**Figure 7:**
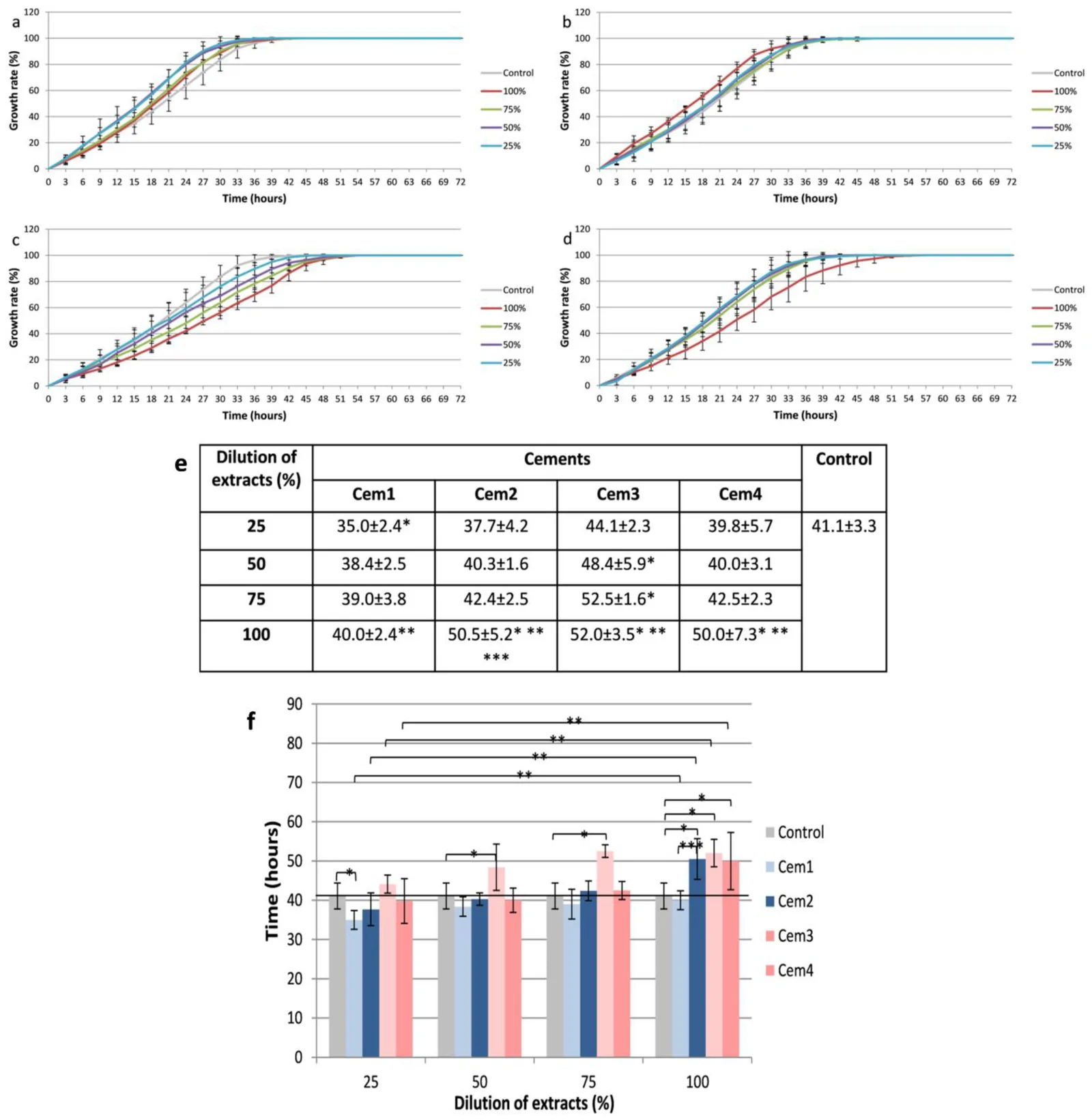
Dynamics of restoration of the monolayer defect of MG-63 cell culture (scratch test), obtained in real time during their growth in extracts: (**a**) 25%, (**b**) 50%, (**c**) 75%, and (**d**) 100% of Cem1, Cem2, Cem3, Cem4 samples; (**e**,**f**) time (hours) of restoration of the monolayer of the MG-63 cell culture depending on the concentration of the extracts Cem1-Cem4 of the samples. *—statistically significant difference between the recovery time of the MG-63 cell monolayer in the control and Cem1-Cem4 groups with different dilutions of extracts (comparisons were made in pairs); **—statistically significant difference between the recovery time of the MG-63 cell monolayer in the experimental groups Cem1-Cem4 between 25 and 100% extracts (comparisons were made in pairs); ***—statistically significant difference between the recovery time of the MG-63 cell monolayer in the experimental groups depending on the magnesium content in the samples: Cem2 vs. Cem1 and Cem4 vs. Cem3.

The lack of toxicity in these cement samples is further confirmed by the results of experiments evaluating the recovery dynamics of an MG-63 cell monolayer using a “scratch” assay (Figure 7a–f). In the control group, the time required for defect closure was 41.3 h. In the experimental groups, this duration depended on the dilution level of the cement sample extracts. When 25% extracts were used, the migration rate of MG-63 cells for Cem2, Cem3, and Cem4 was comparable to that of the control, with monolayer recovery taking 37.7, 44.1, and 39.8 h, respectively; for Cem1, the closure time was the shortest—35.0 h—which was statistically significantly lower than in the control (*p* = 0.003). The use of 50% and 75% extracts of the cement samples resulted in an increase in the time required for defect closure; however, for Cem1, Cem2, and Cem4, this duration did not differ statistically significantly from the control, whereas in the Cem3 extracts, cell migration was significantly slower than in the control (*p* = 0.014 and 0.000, respectively). Finally, the 100% extract had no effect on the defect closure rate for Cem1, but it inhibited the migration of MG-63 cells for Cem2, Cem3, and Cem4, with complete monolayer recovery observed at 50.5, 52.0, and 50.0 h, respectively, compared to 41.3 h in the control (*p* = 0.002, 0.002, and 0.014, respectively). To evaluate the effect of magnesium content on the migration of MG-63 cells, a pairwise statistical analysis was performed on 100% extracts for the groups Cem1 vs. Cem2 and Cem3 vs. Cem4. It was shown that cells migrate significantly faster in the Cem1 extract (20% magnesium; liquid phase: acidic sodium phosphate) than in the Cem2 extract (60% magnesium with the same liquid phase; *p* = 0.001). No significant differences were found between the Cem3 and Cem4 groups (20% and 60% magnesium, respectively; liquid phase: CMC) (*p* = 0.673). When comparing the effect of the liquid phase composition (acidic sodium phosphate vs. CMC) at a fixed magnesium concentration, a statistically significant difference was observed between the Cem1 and Cem3 groups: in the 20% extract prepared with the acidic sodium phosphate-based liquid phase, the defect closed more rapidly than in the extract prepared with CMC (*p* = 0.001). No significant differences were found between the Cem2 and Cem4 groups (*p* = 0.893). Based on these preliminary data, it can be hypothesized that extracts containing 60% magnesium—regardless of whether the liquid phase is CMC or the acidic sodium phosphate-based solution—inhibit the migration of MG-63 cells in a scratch assay; consequently, in the Cem2, Cem3, and Cem4 groups, the restoration of the cell monolayer is delayed by 9–11 h.

**Figure 8:**
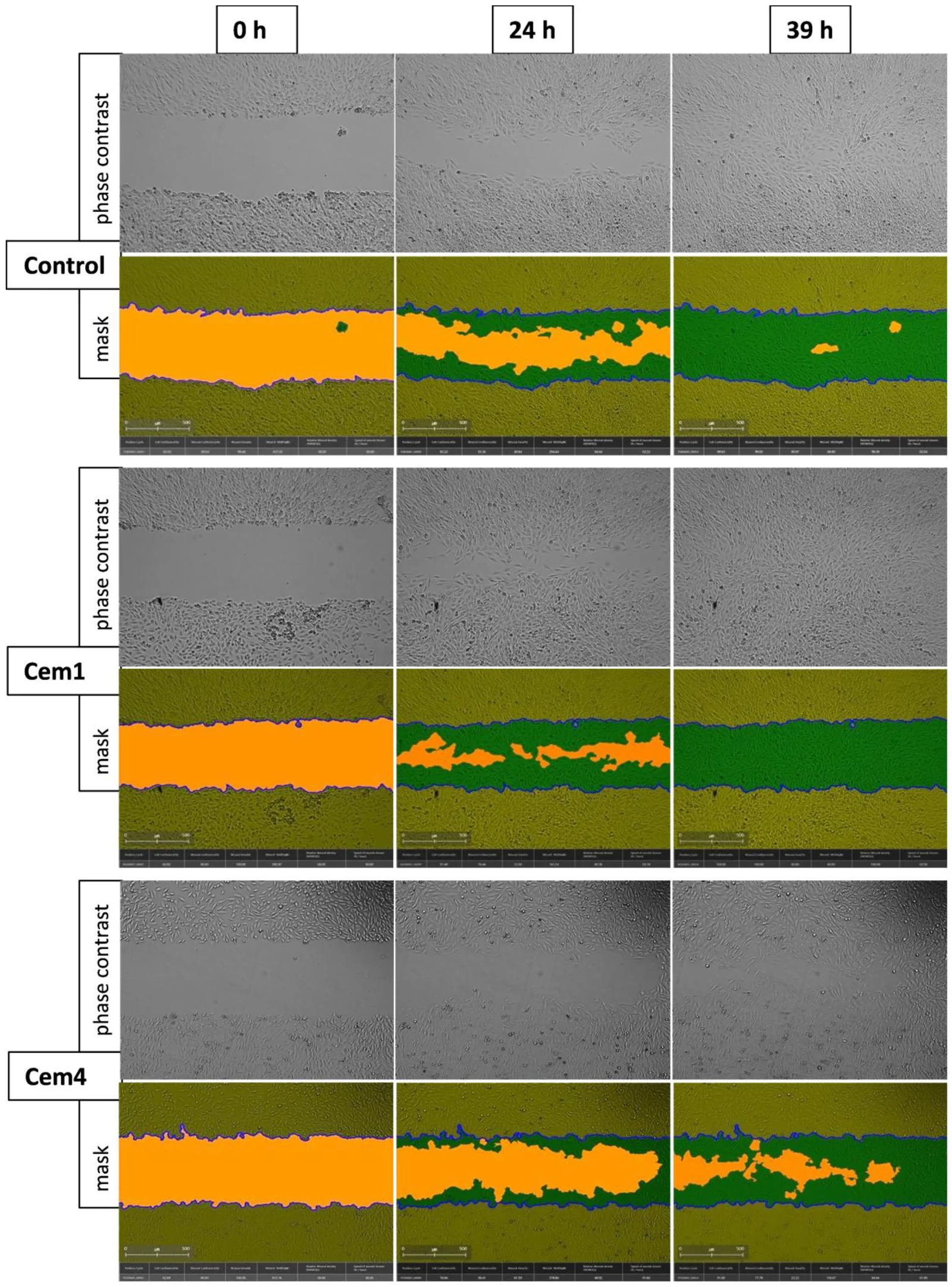
Light microscopy of restoration of the defect area (magnification ×4) (scratch test).

Figure 8 shows the dynamics of monolayer defect recovery in the control and, as an example, for Cem1 and Cem4 samples. It is evident that in the Cem1 sample, the defect had closed just 39 h after the scratch was created, whereas in the control and the Cem4 sample, monolayer formation was not yet complete.

Overall, the results obtained in this section of the study indicate that samples Cem1 through Cem4 exhibit no toxicity toward the MG-63 cell culture.

### 2.7. In Vitro Study of Cytocompatibility of CMPC Samples

To evaluate the cytocompatibility of the developed Cem1-Cem 4 bone cement containing 20 and 60% magnesium, as well as two cement fluid formulations, MG-63 cells were seeded directly onto CMPC samples. Using confocal microscopy, cell attachment to the cement samples and cell spreading were studied in direct contact with the surface of these materials 24 and 72 h after seeding. Furthermore, the metabolic/proliferative activity of MG-63 cells was assessed over the course of the 10-day experiment using the MTT assay, as they grew dynamically on the test cement samples.

**Figure 9:**
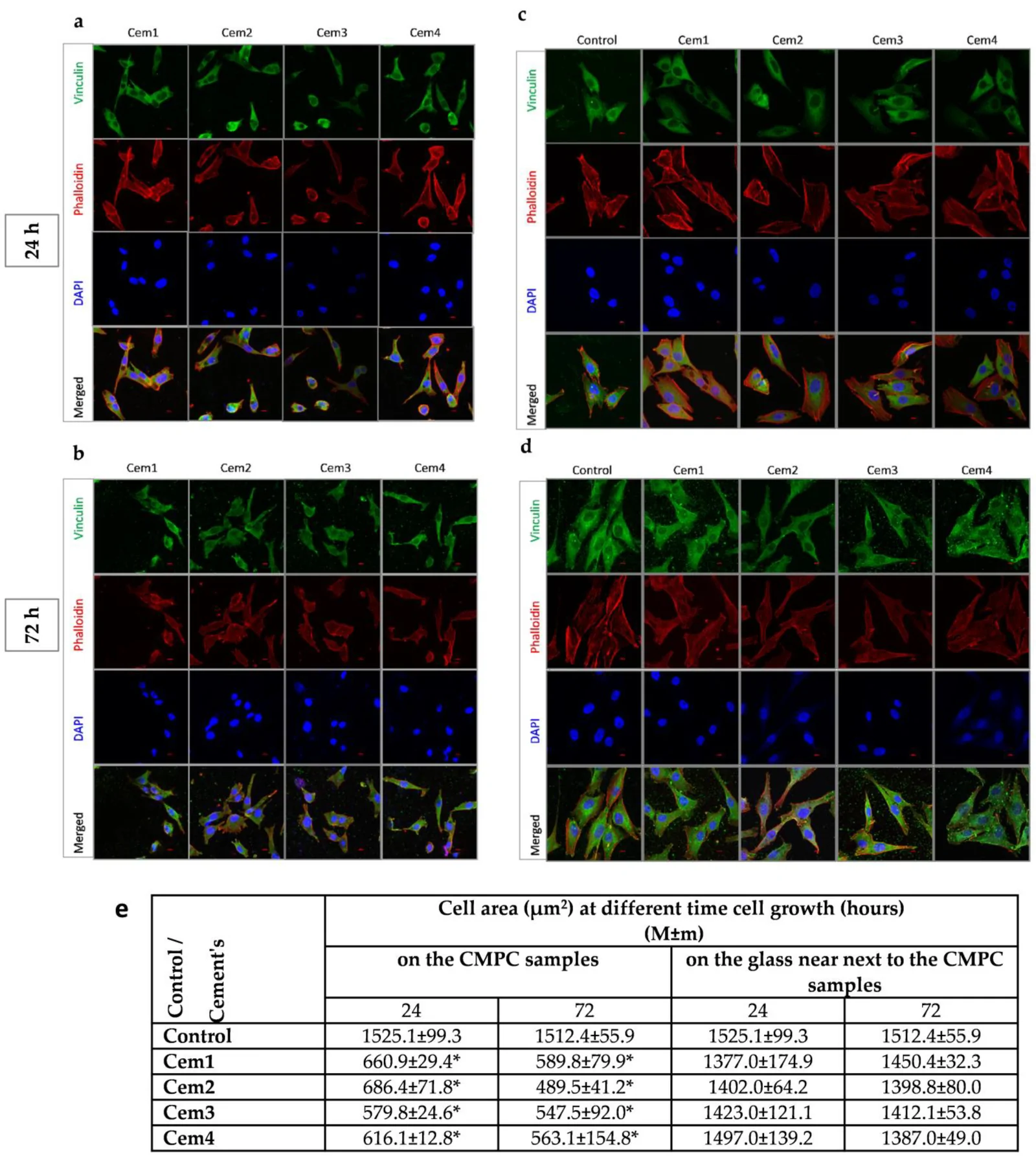
Immunofluorescence images of MG-63 cells after 24 and 72 h of growth: on Cem1, Cem2, Cem3, and Cem4 (**a**,**b**); on culture plastic (control) and on culture plastic near the Cem1, Cem2, Cem3, and Cem4 samples (**c**,**d**), stained with Vinculin (green), Falloidin (red), Dapi (blue), and Merge, scale bar: 10 μm. Area of MG-63 cells on Cem1–Cem4 samples and on polystyrene near the cement samples after 24 and 72 h of cell growth (**e**); *—statistically significant difference compared to the control (*p* < 0.05).

Figure 9a–d shows micrographs of MG-63 cells on Cem1-Cem 4 samples 24 and 72 h after seeding, next to samples on plastic and in the control (plastic) within the same timeframe. It is shown that 24 h after seeding Cem1-Cem4 samples, the MG-63 cell population, regardless of the composition and type of cement liquid, was represented mainly by small spindle-shaped cells with a centrally located nucleus (Figure 9a).

In the control (polystyrene culture plastic), MG-63 cells at this observation period were well spread out, had a polygonal shape with a nucleus in the center (Figure 9c), while the area of the cells statistically significantly exceeded the area of the cells on the experimental cement samples (1525.1 μm2 vs. 578.8÷686.4 μm2, *p* ≤ 0.05) (Figure 9e).

After 72 h of observation in the experiment on the cement samples, along with the spindle-shaped cells, polygonal cells appeared, still smaller in comparison with the control, where the cell population was represented by large flattened polygonal cells with an area of 1512 μm2 vs. 489.5÷589.8 μm2 in the experiment (the difference is statistically significant, *p* ≤ 0.05) (Figure 9b,d,e).

The obtained results on smaller MG-63 cells on experimental cement samples compared to the control 24 and 72 h after their seeding can be explained by the microstructure of their surface compared to absolutely smooth polystyrene.

Observation of the MG-63 cell pool after 24 and 72 h, which was seeded on polystyrene next to the CMPC samples, allowed us to conclude that their shape and size were similar to the control at the specified time points (the difference was not significant, *p*≥ 0.05) (Figure 9c–e). It is noteworthy that after 72 vs. 24 h the area of the MG-63 cell population decreased both in the control and in the experiment (the difference is not significant, *p* ≥ 0.05), which can be explained by an increase in the confluency of the monolayer of this cell culture and, as a result, a surface deficit.

**Figure 10:**
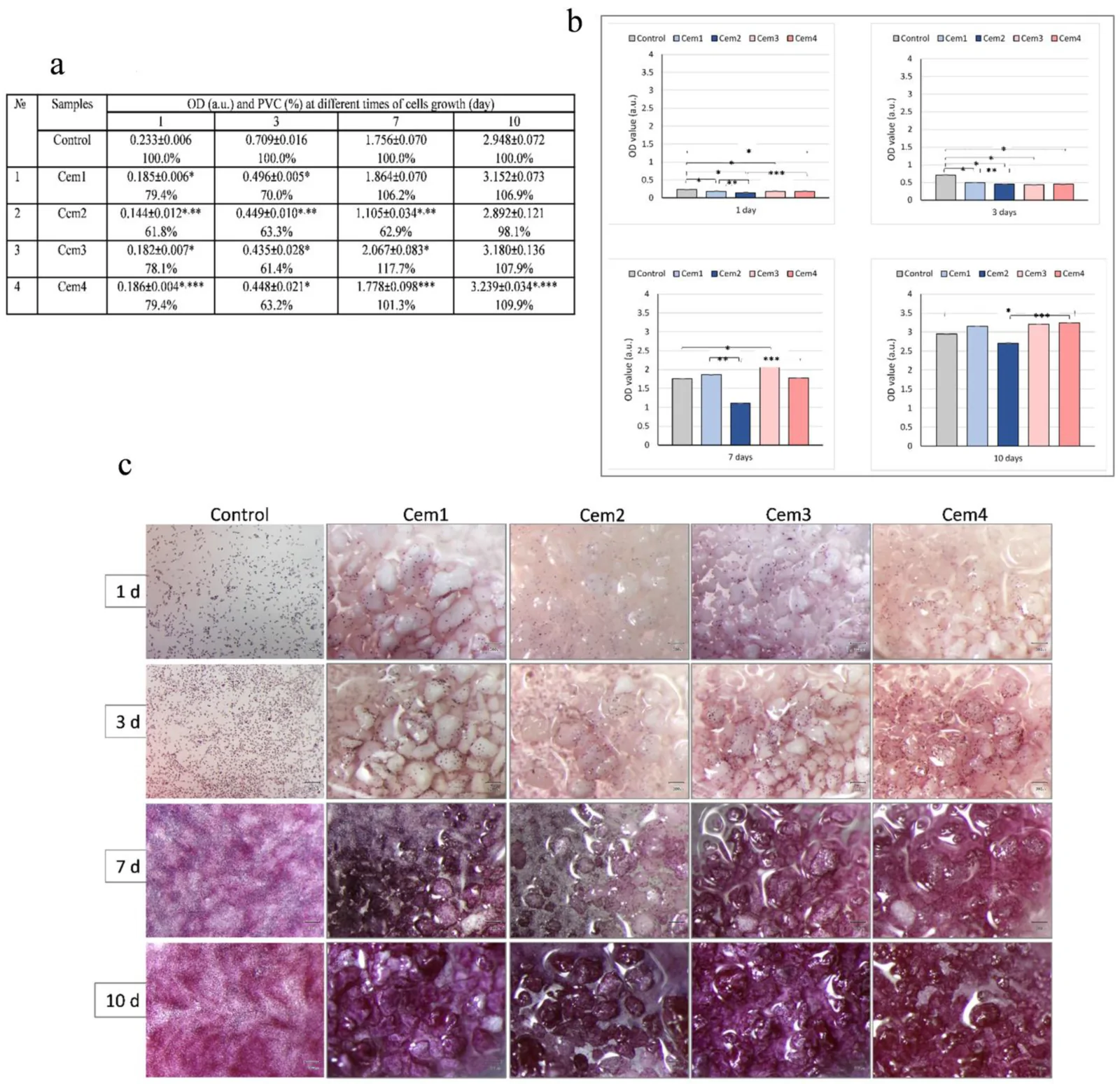
Cytocompatibility of Cem1, Cem2, Cem3, and Cem4 samples (direct test): (**a**) Optical density of formazan solution (OD, MTT-test, mean ± standard error, *n* = 3) and PVC of human osteosarcoma MG-63 cells on polystyrene (control) and experimental cement samples at different incubation times (significant difference determined via Student’s-test (*p* ≤ 0.05): *—between experimental groups and control; **—between experimental groups Cem1–Cem2 and Cem3–Cem4; ***—between experimental groups Cem1–Cem3 and Cem2–Cem4. (**b**) Pool of MG-63 cells on polystyrene (control) and Cem1–Cem4 samples (experiment) at different times of cell growth. (**c**) Expansion of the surface of bone cement samples Cem1, Cem2, Cem3, and Cem4 by MG-63 cells (1, 3, 7, and 10 days of cell growth, light microscopy, MTT-test). Scale bar: 300 μm.

Despite the smaller size of MG-63 cells compared to the control in the early stages after their seeding on the cement samples, they were metabolically active during long-term observation (up to 10 days), which was reflected in their proliferation rate and colonization of the surface of the CMPC samples (MTT test data, Figure 10). In the control, the MG-63 cell pool constantly increased over the entire observation period, as evidenced by the optical density (OD) values of the formazan solution at the stages of the experiment: 0.233 a.u. (1st day), 0.709 a.u. (7th day) and 2.948 a.u. (10th day). In the experiment group, the number of MG-63 cells on all CMPC samples was statistically significantly lower than in the control group on days 1 and 3 of culture. By day 7, the cell populations on samples Cem1 and Cem4 did not differ significantly from the control; the population on sample Cem3 was higher, while only sample Cem2 continued to show a lower cell count compared to the control. By the end of the observation period (day 10), the number of MG-63 cells on the CMPC samples either showed no statistically significant difference from the control (samples Cem1, Cem2, and Cem3) or was significantly higher (sample Cem4, *p* = 0.022; Figure 10a,b).

A pairwise comparison of the growth dynamics of MG-63 cells on CMPC samples containing 20 mol.% and 60 mol.% magnesium (samples Cem1 and Cem2, prepared with cement liquid A), as well as on samples Cem3 and Cem4 (prepared with cement liquid CMC), revealed that cell proliferation was significantly more active on sample Cem1 than on sample Cem2 during the period from day 1 to day 7 (inclusive) (*p* = 0.040, 0.013, and 0.000, respectively). By the 10th day, this difference had leveled out. In the groups using bone cements Cem3 and Cem4, no differences in the population dynamics of MG-63 cells were observed throughout the entire observation period.

When comparing the results based on the composition of the cement liquid (sodium acid phosphate versus CMC), no statistically significant differences were found for the bone cement samples containing 20% magnesium (Cem1 vs. Cem3). However, for the samples with 60% magnesium (Cem2 vs. Cem4), a statistically significant difference (*p* ≤ 0.05) in the number of MG-63 cells was observed compared to the Cem2 group on days 1, 7, and 10 (*p* = 0.027, 0.003, and 0.041, respectively); this indicates that CMC is a more favorable liquid component than sodium acid phosphate. Given the small sample size in the in vitro experiments, it can be suggested that the most promising CMFC formulations are those containing CMC in the mixing liquid, regardless of the magnesium concentration.

Overall, the results obtained in this and previous sections of the study confirm the cytocompatibility of all the developed calcium–magnesium phosphate cement (CMPC) samples. Regardless of the magnesium content or the composition of the liquid component, MG-63 cells attach to the surface of these bone cements and begin to proliferate (though a certain delay is observed during the early stages of growth, as determined by the optical density of the formazan solution and the PVC index). This phenomenon can be attributed to the release of CMPC biodegradation products into the culture medium; regular replacement of the medium in the wells naturally leads to a decrease in the concentration of these products. Nevertheless, active proliferation of MG-63 cells across the surfaces of the Cem1–Cem4 samples was observed in all experimental groups. Nevertheless, in all experimental groups, marked proliferation of MG-63 cells was observed on the surface of the Cem1–Cem4 samples; as a result, by the end of the experiment—on the 10th day of observation—the cells had literally enveloped each cement particle (Figure 10c).

### 2.8. Effect of CMPC on the Osteogenic Differentiation of BM hMSCs

The expression of certain genes involved in osteogenic differentiation was evaluated by RT-PCR. Assays were performed at day 0 for untreated (undifferentiated control) cells and 3 weeks (day 21) after adding the cocktail of osteogenic factors to the mesenchymal stromal cells from donor bone marrow (BM hMSCs) cultured in the presence of Mg-substituted bone cements.

The expression of specific genes was determined and normalized to *GAPDH*. Fold-change (FC) values were calculated relative to the day 0 sample. Among the genes that are important for osteogenesis, we have assessed *RUNX2* (encoding runt-related transcription factor 2), *ALPL* (encoding tissue-nonspecific alkaline phosphatase), and *SP7* (osterix (OSX) transcription factor) expression levels.

**Figure 11:**
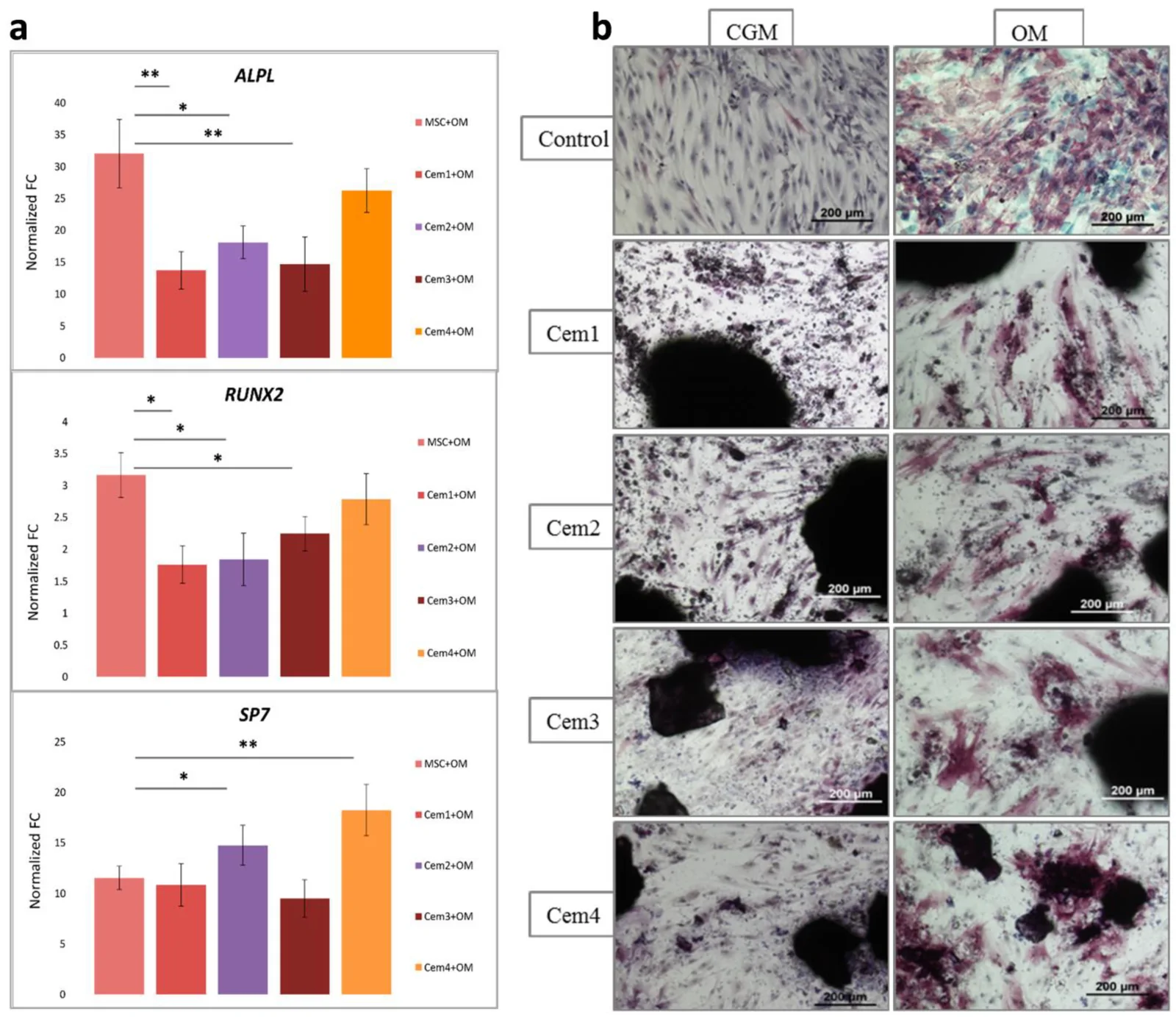
Effect of Cem1, Cem2, Cem3, and Cem4 on BM hMSC in vitro osteogenesis. (**a**) The expression levels of osteogenesis-related genes *ALPL*, *RUNX2*, and *SP7*. Error bars represent means ± SD for *n* = 3. Asterisks indicate statistical significance at *p* < 0.05 (one asterisk) and *p* < 0.01 (two asterisks. (**b**) ALPL staining.

The addition of osteogenic differentiation factors evidently increased the expression of the *ALPL* gene in all induced BM hMSC cultures, but its expression was significantly lower in the presence of bone cements, with the exception of the Cem4 sample, for which the level of ALPL expression did not differ from the control (Figure 11a). The changes in the expression levels of Cem2 and Cem4 were slightly greater compared to other compositions; most appeared due to higher Mg^2+^ concentration, but they did not reach statistical significance compared to the control BM hMSC+OM culture level of specific genes. Similarly, *RUNX2* expression in the presence of Cem1-Cem3 bone cements was lower than in the control (*p* ˂ 0.05) and for the Cem4 sample did not differ from the control, but its levels increased as the concentration of Mg^2+^ rose. Finally, it was found that in the presence of Cem2 and Cem4, the expression level of the SP7 gene was significantly higher compared to the control (*p* = 0.047 and *p* = 0.008, respectively), which could possibly indicate a stimulatory effect associated with a high concentration of magnesium ions.

These RT-PCR results are consistent with the cytological ALPL staining results described above when comparing the composition of Mg-substituted bone cements in terms of their ability to stimulate and maintain BM hMSC differentiation into the osteogenic lineage (Figure 11b).

Taken together, the data indicate that all the bone cement formulations tested (Cem1-Cem4) are capable of supporting the differentiation of BM hMSCs into osteoblasts, with specific gene expression levels being most pronounced in cement samples containing 60 mol.% magnesium.

### 2.9. Biocompatibility of Cem1-Cem4 Sample In Vivo Experiments

**Figure 12:**
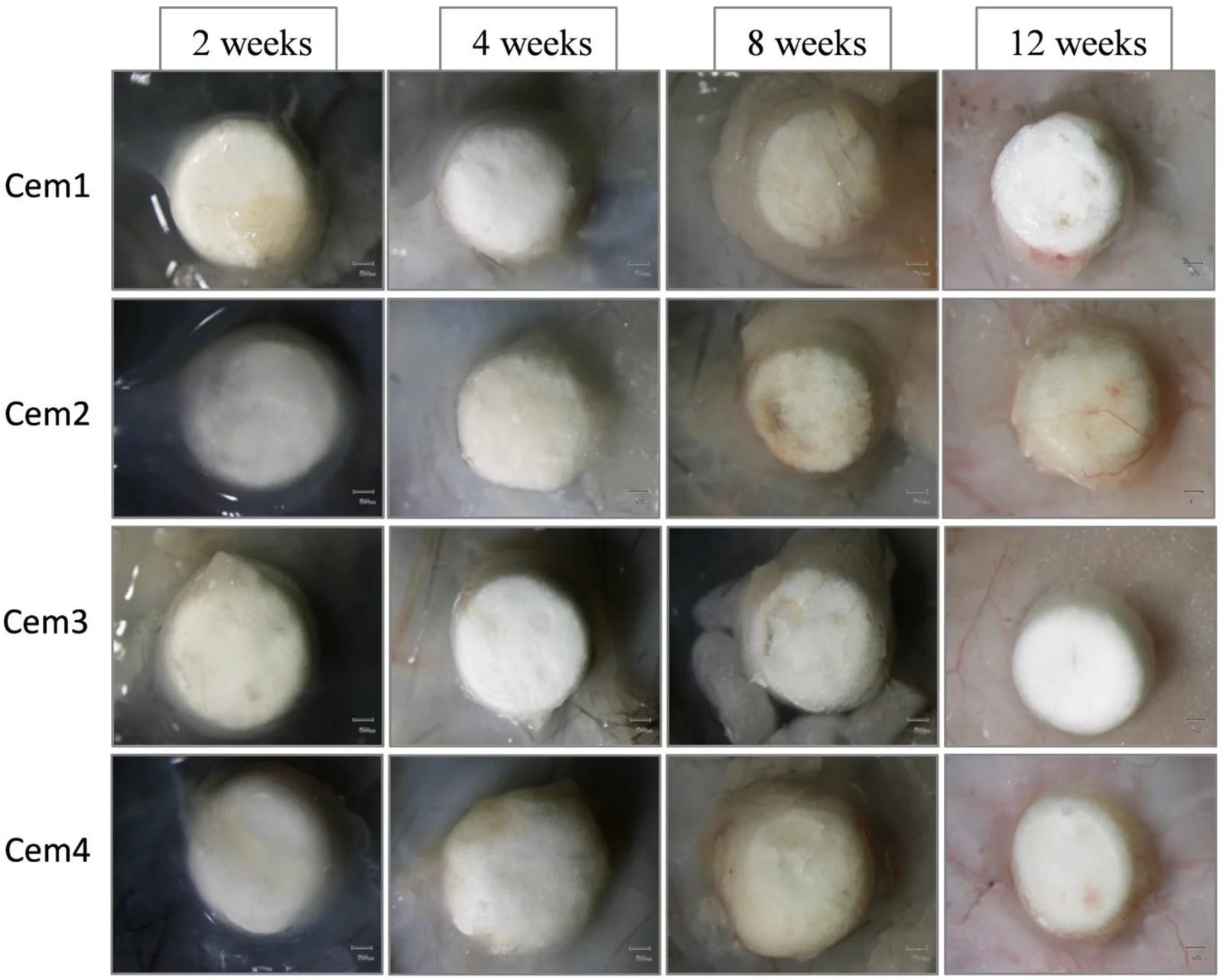
Appearance of Cem1–Cem4 samples after subcutaneous implantation. Scale bars: 500 µm.

The appearance of Cem1-Cem4 samples after their subcutaneous implantation into mice at different observation times is shown in Figure 12. It is evident that all cement specimens are surrounded by a translucent, poorly vascularized connective tissue capsule. Over observation periods of 2–12 weeks, regardless of magnesium content and cement fluid composition, their appearance remains virtually unchanged, reflecting a slight decrease in volume and indicating weak bioresorption of these cement specimens during this time period.

**Figure 13:**
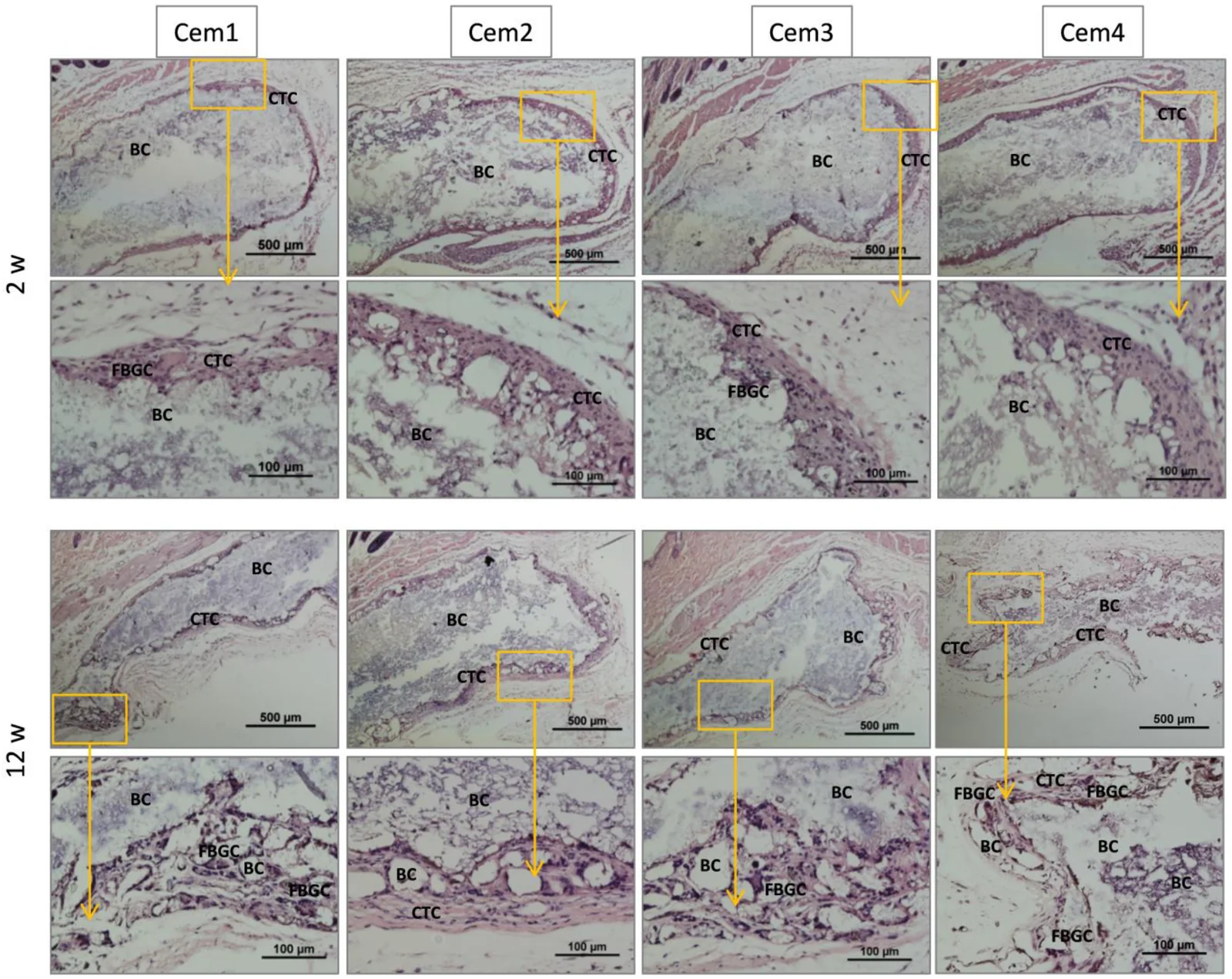
Micrographs of Cem 1–Cem 4 samples obtained after 2 and 12 weeks of their subcutaneous implantation. Stain: hematoxylin and eosin. Scale bars: 500 and 100 µm. Abbreviations: BC—bone cement; CTC—connective tissue capsule; FBGC—foreign body giant cells.

Figure 13 shows histological sections of the CMPC samples after 2 and 12 weeks of observation. It is shown that, 2 weeks after subcutaneous implantation, a well-organized, moderately vascularized connective tissue capsule is formed around the Cem1-Cem4 samples, closely adjacent to them. It is noted that at this observation period, for all experimental samples, the capsule is thinner on the skin side and somewhat thicker on the subcutaneous tissue side and the sides of the implant. The onset of bone cement resorption is indicated by the fistulous nature of their edges and the presence of a few giant foreign body cells. During this observation period, fibroblast ingrowth from the periphery of the cement samples is noted. For the Cem1 and Cem4 samples (20 mol% magnesium, cement fluids-sodium acid phosphate and CMC), no signs of inflammation or rejection were detected at the implantation site at this or subsequent observation periods. Increasing magnesium to 60% in the material leads to the appearance of a weak neutrophilic reaction at the implantation site after two weeks (data not presented) and the absence of such at other stages of the experiment.

After 12 weeks of the experiment, indirect macroscopic signs of biodegradation of the Cem1-Cem4 samples include a decrease in size and a change in shape from a regular oval to an elongated shape with jagged edges. Microscopic signs include the presence of a few FBGC, fragmentation of the cement mass at the periphery, and the “pinching” of fragments due to the ingrowth of connective tissue strands. By 12 weeks after subcutaneous implantation, the bioresorption of the CMPC samples was not complete in either experimental group.

### 2.10. Osteoplastic Potencies of Cem1-Cem4 Samples According to In Vivo Experiments

Following the creation of a bone defect—specifically a marginal resection of the rat tibia—and its closure with samples Cem1 through Cem4, no postoperative complications were observed in any of the operated animals; wounds healed by primary intention, and all animals remained active and mobile with normal appetites. Macroscopic examination of tissue samples harvested from the defect site at 3, 9, and 12 weeks, as well as 6 months post-operation, revealed no signs of inflammation.

**Figure 14:**
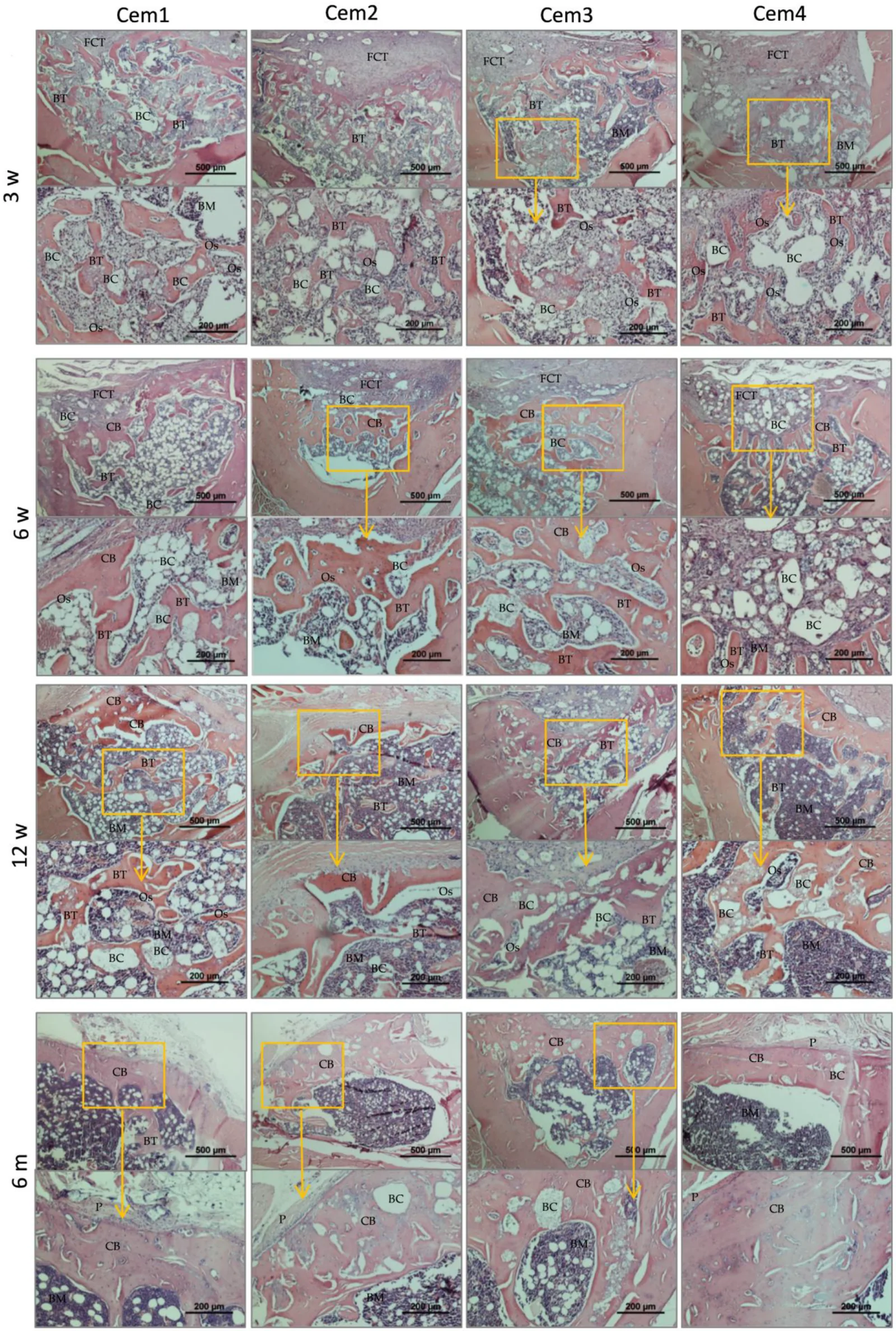
Micrographs of Cem1-Cem4 samples obtained at 3, 6, 12 weeks and 6 months after their implantation into a bone defect of the rat tibia. Stain: hematoxylin and eosin. Scale bars: 500 and 100 µm. Abbreviations: BC—bone cement; BT—bone trabeculae; FCT—fibrous connective tissue; BM—bone marrow; Os—osteoblast; CB—cortical bone; P–periosteum.

Histological sections obtained 3 weeks after defect closure showed good bonding between the cement masses and the host bone bed, with intimate apposition and no intervening connective tissue (Figure 14). Within the defect zone, the implant no longer appeared as a monolithic cement mass but rather as numerous fragments of varying sizes, situated either amidst newly forming bone trabeculae or already incorporated into their structure. At this stage, the bone regenerate consisted of numerous trabeculae of immature coarse-fibered bone tissue forming across virtually the entire area of the defect. Reparative osteogenesis was highly active during this period, evidenced by numerous microscopic fields showing chains of active osteoblasts lining the exterior of the newly formed bone trabeculae. A small proportion of the cement fragments had become incorporated into the structure of the bone trabeculae. Externally, the bone defect was covered by a broad rim of fibrous connective tissue containing numerous blood vessels and small fragments of bone cement; the presence of abundant foreign-body giant cells (FBGCs) in the vicinity of these fragments indicated active resorption. Six weeks after the implantation of Cem1–Cem4, the bone defect is not yet completely closed; the bone regenerate consists of newly formed trabeculae containing remnants of the cement material. Reparative osteogenesis is highly active, as evidenced by the presence of numerous chains of osteoblasts on the surfaces of the bone trabeculae. Bone marrow hematopoiesis is being restored. The defect is covered externally by a layer of moderately vascularized fibrous connective tissue—denser than that observed at the previous time point—which contains scattered cement particles and numerous multinucleated foreign-body giant cells (FBGCs).

By the 12th week of the experiment, all experimental groups showed a restoration of bone marrow hematopoiesis and active de novo osteogenesis originating from the recipient’s bone bed within the defect zones. This was evidenced by the formation of numerous new bone trabeculae containing inclusions of non-resorbed cement fragments and chains of active osteoblasts. At this stage, the bone defect was closed; however, a fully developed cortical plate did not form in any of the groups. On the outer aspect, in groups Cem3 and Cem4, the defect was covered by a thin yet sufficiently dense layer of fibrous connective tissue containing residual cement fragments and numerous multinucleated giant cells, whereas in groups Cem1 and Cem2, the defect was covered by a newly formed periosteal layer (Figure 14).

Six months post-surgery, the bone defect in animals from all groups was completely closed by a formed cortical plate containing Haversian canals. During this period, the medullary canal and bone marrow hematopoiesis were restored. Externally, the defect was covered by a dense periosteum with a sparse network of blood vessels. Isolated inclusions of residual Cem2 and Cem3 materials were noted within the cortical plate (Figure 14).

Overall, the study of the osteoplastic potential of CMPC samples with magnesium contents of 20 and 60 mol% indicates that all of them—regardless of magnesium content or cement liquid composition—possess osteoconductive properties. Notably, their biodegradation rate is comparable to the rate of new bone tissue formation. Thanks to these properties, organotypically mature bone tissue had formed at the implantation site six months after the Cem1–Cem4 samples were placed in the rat tibial defects.

### 2.11. Micro-CT Analysis

The bone volume fraction (BV/TV) was calculated from micro-CT data for the control group (defect without cement) at 6 weeks, and for the Cem1–Cem4 groups at 6 weeks. Data are presented as BV/TV and as a percentage (Table 3).

In the control group, BV/TV reached 0.179 (17.9%) at 6 weeks, which is typical of a phase dominated by resorption and remodeling rather than active bone formation.

In the bone cement groups, BV/TV for Cem1–Cem3 fell within a similar range of approximately 17% (0.163, 0.168, and 0.165, respectively), with no significant differences between these groups at 6 weeks. Cem4, which was formed by powders with 60 mol.% Mg and the cement liquid contained polymer CMC, demonstrated a significantly higher value—0.214 (21.4%). The mean VOI density followed a similar trend: 204.9–209.6 mg HA/cm^3^ for Cem1–Cem3 and 253.3 mg HA/cm^3^ for Cem4. These findings suggest that application of Cem4 leads to an increase in bone tissue of +19.6% at 6 weeks compared with the control group. The higher magnesium content in Cem4 appears to stimulate new bone tissue formation, which may be attributed to the well-documented osteogenic and osteoconductive effects of magnesium ions.

**Figure 15:**
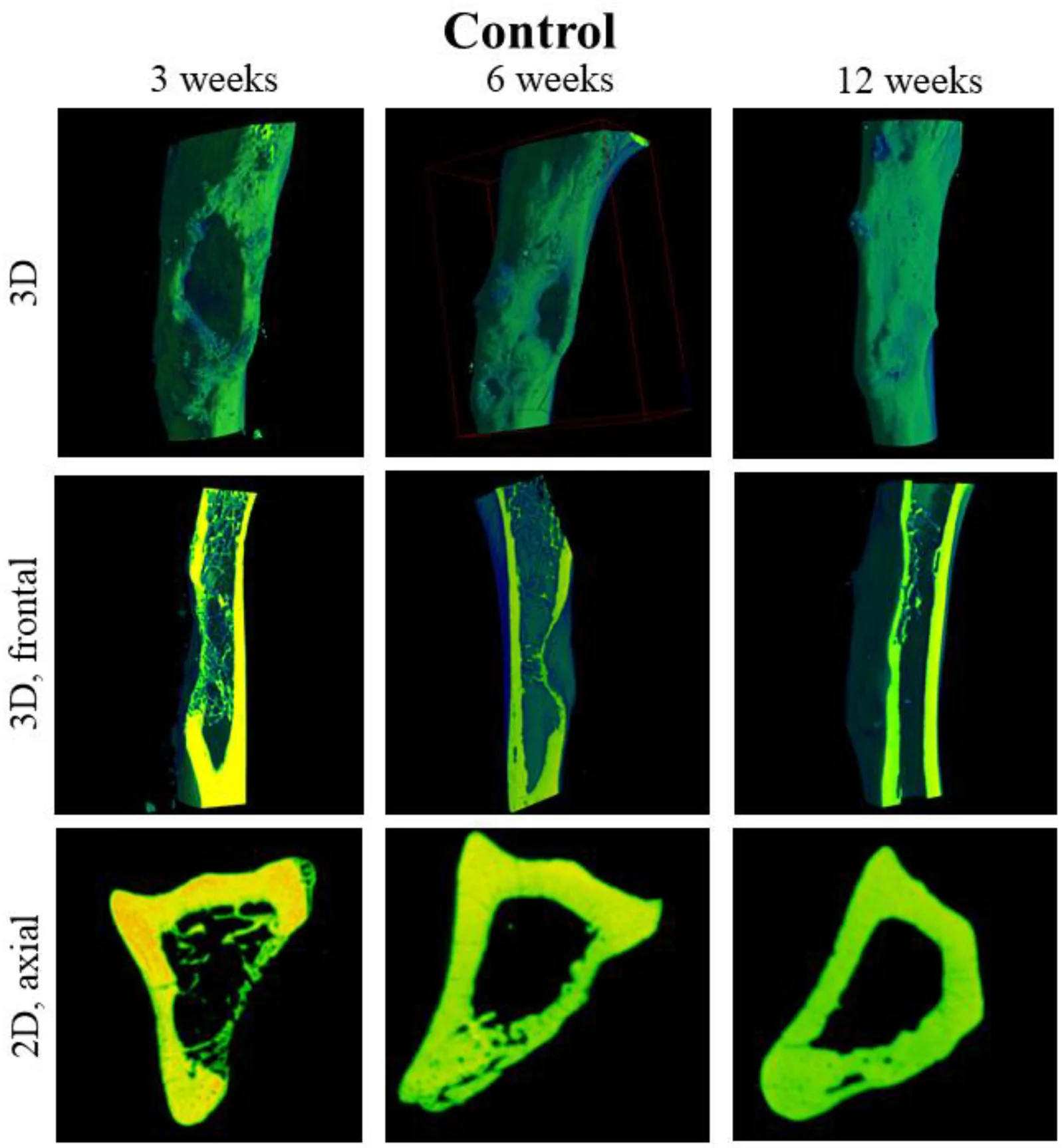
Representative micro-CT visualization of the control group (defect without cement) at 3, 6, and 12 weeks. Three-dimensional reconstruction (**top row**), 3D frontal section (**middle row**), and 2D axial section (**bottom row**) are shown for each time point. Only mineralized bone tissue is displayed. The color gradient indicates bone density, ranging from blue (lower density) to green (higher density). At 12 weeks, the bone defect is completely restored with the formation of dense cortical bone.

Representative micro-CT images of the control group (defect without cement) at 3, 6, and 12 weeks are shown in Figure 15. Three-dimensional reconstruction, 3D frontal section, and 2D axial section are presented for each time point, with only mineralized bone tissue displayed. The color gradient reflects bone density, ranging from blue (lower density) to green (higher density).

At 3 weeks, the defect area was partially filled with newly formed bone of low density, predominantly located at the defect margins, while the central region remained largely unmineralized. By 6 weeks, a slight increase in bone density was observed within the defect, although the central region was still not fully restored. At 12 weeks, the defect was completely filled with dense mineralized bone, and the cortical layer was fully restored, as clearly seen in both the 3D reconstruction and the 2D axial section. The color distribution shifted toward green, indicating a higher degree of mineralization compared with earlier time points.

**Figure 16:**
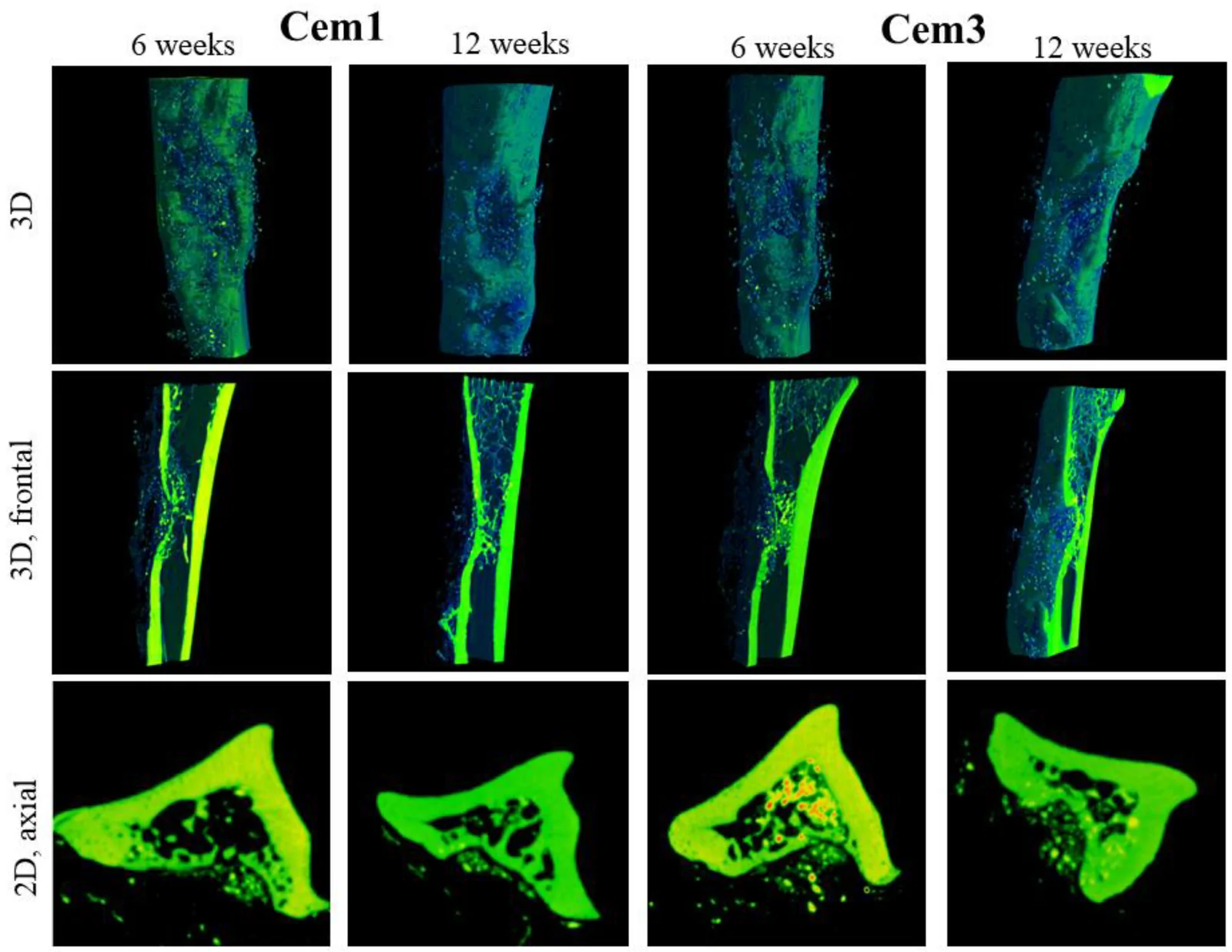
Representative micro-CT visualization of the Cem1 and Cem3 groups at 6 and 12 weeks. Three-dimensional reconstruction (**top row**), 3D frontal section (**middle row**), and 2D axial section (**bottom row**) are shown for each time point. Only mineralized bone tissue is displayed. The color gradient indicates bone density, ranging from blue (lower density) to green (higher density).

Representative micro-CT images of the Cem1 and Cem3 groups at 6 and 12 weeks are shown in Figure 16. In both Cem1 (containing 20 mol.% Mg) and Cem3 (containing 20 mol.% Mg and CMC polymer) groups, incomplete adaptation of the cement material to the bone bed was observed. Washout of cement from the defect area occurred, with cement granules localized outside the defect margins. At 6 weeks, a thin bone septum was formed within the defect area. By 12 weeks, increased new bone formation and partial closure of the defect were observed; however, the defect was not completely restored. The color distribution in the 12-week images showed a shift toward green, indicating a higher degree of mineralization compared with 6 weeks, although the defect volume remained incompletely filled.

**Figure 17:**
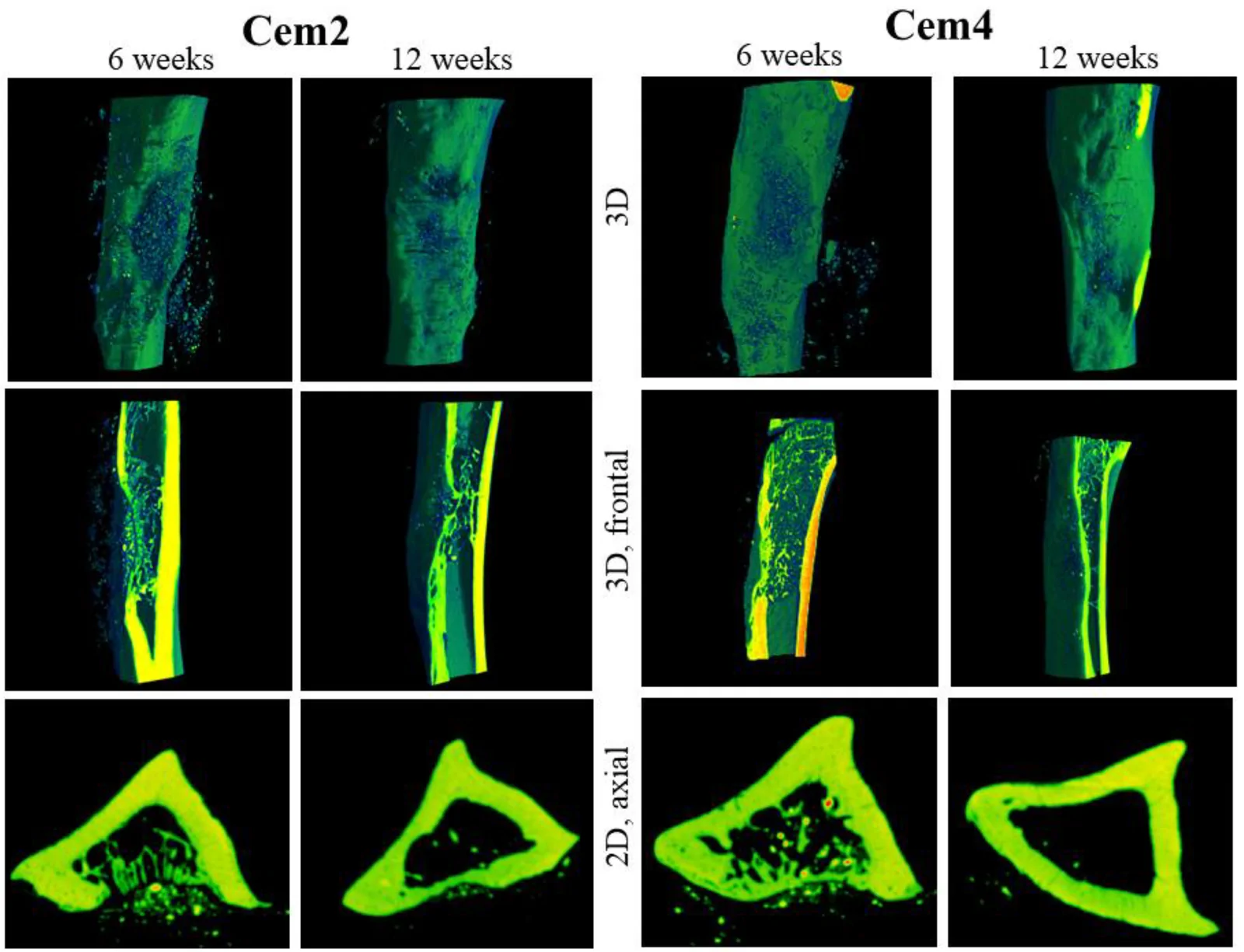
Representative micro-CT visualization of the Cem2 and Cem4 groups at 6 and 12 weeks. Three-dimensional reconstruction (**top row**), 3D frontal section (**middle row**), and 2D axial section (**bottom row**) are shown for each time point.

Micro-CT images of the Cem2 and Cem4 groups at 6 and 12 weeks are shown in Figure 17. Both groups contain 60 mol.% Mg; Cem4 additionally contains CMC polymer. In the Cem2 group, partial washout of cement particles into the surrounding tissues was observed at 6 weeks. At this time point, the formation of the cortical wall began. By 12 weeks, a denser bone structure had formed, and the defect was almost completely restored with restoration of volume. In the Cem4 group, less cement leakage from the defect area was observed compared with Cem2. At 6 weeks, active formation of a bone septum was noted. By 12 weeks, the defect was completely closed, with restoration of the cortical volume of the tibia. The color distribution in the 12-week images shifted toward green, indicating a higher degree of mineralization compared with 6 weeks. Accelerated defect restoration was observed in the Cem4 group as early as 6 weeks compared with the control group, as confirmed by the morphometric data. The defect restoration rate in the Cem4 group was approximately twice as fast as in the control group: BV/TV values achieved by the control group at 12 weeks (0.220) were reached by the Cem4 group as early as 6 weeks (0.214).

Gradual resorption of the cement material was observed in all groups (Cem1–Cem4) between 6 and 12 weeks, as evidenced by a decrease in the volume of radiopaque cement particles. While new bone tissue formation occurred in all groups, the rate of defect closure differed among them. The incorporation of the polymer improves the cohesion of the cement paste, thereby reducing washout of the bone cement from the defect area and the release of magnesium ions promotes the acceleration of osteogenesis.

## 3. Discussion

The challenge of identifying implants to repair bone defects—whether resulting from the surgical removal of primary tumors or metastases, trauma, or degenerative diseases—remains a pressing issue. This is primarily due to the slow bioresorption rates of conventional synthetic calcium-phosphate biomaterials (such as ceramics and bone cements), which delay neo-osteogenesis and lead to the formation of bone tissue containing residual, unresorbed implant material at the defect site [55]. Thus, alongside mechanical strength, cytocompatibility, biocompatibility, and osteoconductive/osteoinductive potential, a key requirement for new osteoplasty biomaterials is their ability to undergo bioresorption—a process that ideally should match the rate of neo-osteogenesis [90]. In this regard, researchers and clinicians place high hopes on next-generation biomaterials that incorporate the bioactive element magnesium [54,91,92,93].

Several evident advantages of Mg-substituted CPCs—including enhanced osteoinductive properties and controlled bioresorption kinetics—make them promising materials for dental implantology and maxillofacial surgery [94,95]. In particular, such materials can be used for alveolar ridge augmentation to fill bone volume deficiencies in extraction sockets, thereby ensuring stable anchorage for future dental implants [96,97]. Modifying adhesive properties through the addition of biomimetic polymers could significantly improve the efficacy of craniofacial defect repair, where Mg-substituted CPCs can be employed to fill contours and non-load-bearing structural voids resulting from trauma, cyst removal, or tumor resection in areas such as the forehead, orbits, and mandible [98]. Furthermore, emerging bone cement formulations are being utilized as resorbable adhesives to temporarily stabilize fragmented or comminuted bone segments prior to definitive fixation with hardware [95]. Antimicrobial properties serve as an additional factor favoring the use of magnesium-substituted bone cements in oral surgery, a field often characterized by contamination with pathogenic bacterial flora [99]. At the same time, it is worth noting a number of still unresolved issues regarding the widespread clinical use of such materials—primarily the exothermic effects associated with cement setting and, even more importantly, insufficient mechanical properties—which limit their application to non-load-bearing or minor load-bearing zones of the face [96].

The results obtained in our work demonstrate a relationship between the influence of magnesium concentration, CMC content, cement powder particle size, and the resulting performance properties of the MCPC materials. The formation of newberyite MgHPO_4_·6H_2_O, along with minor amounts of brushite CaHPO_4_·2H_2_O phases was evaluated in the set cement materials (Cem1-Cem4) according to XRD analysis. The dominant phases based on initial cement powders in Cem2 and Cem4 were stanfieldite, whereas Mg-whitlockite prevailed in Cem1 and Cem3. These findings are consistent with the acid–base reaction mechanism between the calcium–magnesium phosphate phases and the NaH_2_PO_4_ based cement liquid, where the release of Ca^2+^ and Mg^2+^ ions leads to the precipitation of brushite and newberyite, respectively.

The incorporation of CMC into the cement liquid of the developed CMPCs resulted in several notable changes in the FTIR and microstructure state. The absence of chemical crosslinking between CMC and the cement liquid, evidenced by the lack of gelation or turbidity upon mixing, further supports this conclusion. The FTIR spectra of CMC-containing samples revealed broadening of the phosphate bands (especially ν3 and ν4), indicating a higher proportion of amorphous or poorly crystalline material. This was attributed to the adsorption of CMC onto the surface of growing crystals through electrostatic interactions between the carboxylate groups (-COO-) of the polymer and Ca^2+^/Mg^2+^ ions, as well as hydrogen bonding between hydroxyl groups and phosphate ions. These interactions effectively inhibited anisotropic crystal growth, resulting in smaller, more rounded crystallites and reduced crystallinity. This behavior is in good agreement with previous studies on polysaccharide-modified calcium phosphate cements, where polymers such as CMC and polyacrylic acid were shown to refine crystal structure without participating directly in the phase’s formation [100]. Thus, when anionic polysaccharides are introduced into CPCs, their primary role is to act as structuring agents and rheology modifiers rather than as reactive components [101].

The retardation of setting time upon CMC addition can be explained by two main mechanisms. First, the presence of the polymer in the cement liquid reduces the mobility of ions required for the nucleation and growth of newberyite crystals [102]. Second, CMC molecules adsorb onto the surface of cement particles, forming a polymer layer that partially blocks reactive sites and delays the dissolution–precipitation process [103]. This effect is beneficial for clinical applications, as extended setting times improve handling and injection windows [104]. No exothermic reaction was observed during the mixing of the cement pastes; the temperature was in the range of startup 30–25 °C and decreased after 10 min to 23 °C.

The effect of CMC on strength is a more complex process. On the one hand, the formation of a polymer film and calcium binding can slow down crystallization and, in some cases, lead to a reduction in early-age strength [105,106]. On the other hand, our data show that, with proper concentration selection, CMC can contribute to a slight increase in long-term strength. The moderate increase in compressive strength up to 10% suggests that CMC does not act as a reinforcing agent per se, but rather improves cohesion within the cement matrix. The polymer chains may bridge the crystalline phases (newberyite, stanfieldite or Mg-whitlockite) and the amorphous polymer phase, thereby enhancing interparticle bonding and distributing localized stress more uniformly. This is likely due to densification of the microstructure through pore filling by the polymer and the formation of additional bonds between hydration products, making the material more cohesive [107,108]. Studies also show that excessive polymer concentration can lead to agglomeration, weakening the structure [109].

For successful passage through narrow needles (e.g., 22 G), the cement paste must be not only flowable but also homogeneous [110]. The most notable improvement was observed in injectability. For Cem1, the addition of CMC raised injectability from 20% to 63–65%. This enhancement is primarily due to the shear-thinning (pseudoplastic) behavior of CMC solutions, which reduces viscosity under applied extrusion pressure while maintaining structural integrity at rest. In addition, the polymer acts as a lubricant, decreasing internal friction between particles during injection. During injection, the extruded paste has to keep its homogeneity; this is a key parameter of practical application for injectable bone cements. For Cem4, injectability improved from <5% to 50% with CMC addition, but did not reach the same level as Cem3. This indicates that the positive effect of the polymer is partially offset by the larger particle size (D90 = 57.6 µm), which increases interparticle friction and hinders flow through narrow injection needles. Pastes based on coarse particles are prone to filter pressing—separation of the liquid phase from solid particles, leading to needle clogging and a sharp drop in injectability [111,112,113]. The addition of polymers, such as alginate [114], chitosan [115], polyhydroxy PEGylatedpoly(glycerol sebacate) [116], and CMC [117], can improve this parameter, but their effectiveness is limited. In contrast, our results demonstrate that CMC at 0.75 wt.% provides a more substantial improvement in injectability, reaching 63–65%. To further increase injectability, a sufficiently fine powder is recommended, which ensures better paste homogenization, combined with the application of polymers. The injectability values reported here are protocol-dependent rather than absolute, since several testing parameters directly affect extrusion. The 3 mm aperture governs flow resistance (approximately proportional to 1/r^4^ according to the Hagen–Poiseuille relation) and is wider than the 1–2 mm needles or 14–18 G cannulas commonly used in injectability tests, so absolute values are not directly comparable with such studies and serve only to rank formulations tested under identical conditions. The piston rate of 1 mm/s (~4.7 mL/min) defines the shear rate: higher rates increase viscous resistance and may promote filter-pressing, whereas lower rates allow more time for viscosity development and early setting. The interval between mixing and testing is also critical because paste viscosity evolves continuously after mixing; a fixed interval was therefore maintained for all samples so that comparisons reflect differences in powder-to-liquid ratio (PLR) rather than in paste maturation. Finally, the 1 mL volume and syringe geometry determine the paste–wall contact area and hence the friction contribution to the recorded force, and because part of the paste inevitably remains on the syringe walls and in the outlet, the calculated value represents the fraction of the loaded mass extruded under the specified conditions rather than a theoretical maximum; injectability was therefore compared only among samples tested with the same syringe, aperture, piston rate, and mixing-to-testing interval.

The promoting dissolution behavior of magnesium phosphate cements (MPC) is a critical factor for their application in bone repair, as it directly influences mechanical stability and the local physiological environment [118,119]. On the other hand, CPC materials exhibit restrictive dissolution behavior, which also undeniably affects the process of osteogenic differentiation and the replacement by newly formed bone tissue. Our results demonstrate that the introduction of CMC into the CMPC has only a minor effect on the overall dissolution profile, a finding that warrants detailed discussion in the context of known mechanisms and is directly related to the amount of newberyite formed in the cement materials. The dissolution process, as observed through weight loss, pH, and ion release, reflects a complex interplay of dissolution and precipitation. The slight pH decrease from 7.2 to 6.4 during the first 7 days is consistent with the active release of Na^+^ ions from the unreacted cement liquid. Crucially, the subsequent release of Mg^2+^ throughout the experiment indicates the gradual dissolution of the newly formed setting phase, newberyite. This is chemically plausible, as newberyite exhibits higher solubility (Ksp = 1.5 × 10^−6^) compared to other magnesium phosphate phases such as struvite (Ksp = 7.1 × 10^−14^) [120,121,122], making it more susceptible to hydrolytic degradation in an aqueous environment such as SBF. The key finding is that, despite the significant impact of CMC on the rheological properties of the cement liquid (as described earlier), its effect on the degradation process is minimal. The overall weight loss for all samples was in a similar range of 11–14%, and the Mg^2+^ release profiles were broadly comparable among the compositions. This suggests that CMC, at the concentrations used (up to 1 wt.%), does not chemically interfere with the primary dissolution reactions of the cement phases.

Quantitative ICP-MS analysis further demonstrated prolonged Mg^2+^ release from all cement formulations during 28 days of immersion in SBF (Figure 4e). The Mg^2+^ concentration reached approximately 35–43 ppm after 21 days and 32–39 ppm after 28 days, with Cem4 exhibiting the highest concentration at day 21 (approximately 43 ppm). However, no consistently higher Mg^2+^ concentration was observed for Cem4 throughout the entire immersion period. These findings indicate that the Mg^2+^ concentration in the surrounding solution is not determined solely by the initial magnesium content of the cement but may also depend on phase composition and the balance between dissolution and secondary precipitation. Importantly, the in vitro dissolution behavior should not be directly equated with biological resorption in vivo, where cellular activity, local fluid dynamics, and tissue remodeling may additionally influence material degradation.

The formation of a CaP layer on the surface of cement materials upon exposure to physiological fluids is a critical indicator of their bioactivity and potential for osseointegration. When calcium phosphate cements (CPCs) are immersed in phosphate-containing solutions such SBF, a typical sequence of surface reactions occurs, leading to the precipitation of a bone-like apatite layer. For example, when brushite is immersed in SBF, the surface layer can transform to apatite within days through a dissolution–precipitation reaction. This apatite layer is chemically and structurally similar to the mineral phase of natural bone, which accounts for the excellent biocompatibility and osteoconductivity of these materials [123].

Thus, the effect of CMC on cement material properties is the result of a synergistic interaction between the polymer and particles, mediated by adsorption and complexation. The key effects include setting retardation and improved injectability. However, the outcome is largely dependent on the dispersity of the starting powder: finer particles enhance both the adsorption effects of the polymer (retarding setting) and provide the foundation for achieving high injectability.

Biological evaluation of the developed cement compositions—containing 20% or 60% magnesium in the cement powders and mixed with sodium acid phosphate and CMC-based liquids—demonstrated an absence of toxicity toward MG-63 cell cultures in vitro (indirect test) and cytocompatibility upon direct seeding of cells onto the Cem1–Cem4 samples (direct test). Confocal microscopy, performed 24 and 72 h after seeding MG-63 cells directly onto the Cem1–Cem4 samples, revealed that the cell surface area was statistically significantly smaller than that of the control group on polystyrene. These findings are consistent with our previous results [72], as well as those reported by Sopcak, T., Medvecky, L., Giretova, M., et al. [124], and they warrant further investigation into the microstructure of the Cem1–Cem4 samples in the context of in vitro cellular growth dynamics. Notably, when MG-63 cells were seeded directly onto the developed cement samples, they retained their functional activity: throughout the 10-day experiment, the cells proliferated and spread across the surface of the cement particles. By the end of the observation period, the MG-63 cell population on the Cem4 sample was statistically significantly larger than that of the control, whereas the populations on the Cem1–Cem3 samples did not differ from the control. In experiments evaluating their influence on the osteogenic differentiation of donor BM-MSCs, it was shown that the expression levels of key osteogenic differentiation genes ALPL and RUNX2 were significantly lower when cultured on Cem1–Cem3, while they did not differ substantially from the control for the Cem4 sample. Conversely, the expression of the SP7 (Osterix) gene was statistically significantly higher in the Cem2 and Cem4 samples.

All developed formulations (Cem1–Cem4) are biocompatible. A minor neutrophilic response, observed at early stages in the experimental groups Cem2 and Cem4 (containing 60 mol% Mg powder), may be attributed to a local reaction of the surrounding tissues to the magnesium-rich cement compositions; however, the underlying mechanism remains unclear, as blood magnesium concentrations in the animals were not measured in this study.

Experiments involving the repair of bone defects (marginal resection of the tibia in Wistar rats) using freshly mixed bone cement samples (Cem1–Cem4) demonstrated the osteoconductive properties of these materials. Histological analysis revealed the absence of a connective tissue layer at the bone cement–host tissue interface across all groups and observation periods, as well as tight apposition of the Cem1–Cem4 samples to the host tissue. Furthermore, *de novo* formation of bone trabeculae was observed throughout the defect volume starting three weeks post-surgery, with numerous fields of view showing active osteoblasts. The participation of Cem1–Cem4 samples in neo-osteogenesis was evidenced by their gradual replacement with newly formed bone tissue over the course of the study; however, because the bioresorption rate of Cem1–Cem3 samples lagged slightly behind new bone formation, isolated fragments of these materials were still detectable in histological sections after six months. Sample Cem4 demonstrated the most optimal balance between bioresorption and neo-osteogenesis: six months post-surgery, organotypically mature bone tissue had formed, complete with a marrow cavity and restored hematopoiesis.

Micro-CT results confirm these findings. The application of Cem4 leads to a significant increase in bone tissue formation at the early stage compared with the control group and Cem1–Cem3 samples and results in complete closure of the bone defect after 12 weeks, accompanied by restoration of the cortical bone volume of the tibia. The incorporation of CMC improves the cohesion of the cement paste, thereby reducing the washout of bone cement from the defect site, while the release of magnesium ions promotes osteogenic differentiation and enhances the osteoconductive properties of the material.

It is important to note that the biological effects of magnesium associated with the enhancement of osteogenesis and angiogenesis manifest with varying degrees of intensity—both in vitro and in vivo—in biomaterials such as magnesium-substituted calcium phosphate ceramics and 3D constructs [125]; magnesium-containing alloys and coatings [126,127]; magnesium phosphate cements [91,128] and calcium–magnesium phosphate bone cements [50].

The choice of magnesium for incorporation into new bone-grafting materials is no coincidence: the Mg^2+^ ion is the fourth most abundant element in the human body and plays a crucial role in bone remodeling and regeneration [129]. Due to their tendency to undergo bioresorption, magnesium-based biomaterials are considered bioactive. Indeed, magnesium enhances osteogenesis and angiogenesis, stimulates osteoblast adhesion and motility, and possesses immunomodulatory properties [127,129].

The comparative evaluation of the four cement formulations provides an opportunity to distinguish the effects associated with magnesium content and CMC incorporation. Increasing magnesium content in cement powders from 20 to 60 mol.% was accompanied by improved compressive strength but reduced injectability of the cements,, whereas the addition of CMC substantially improved injectability at both magnesium concentrations. These observations suggest that magnesium content and CMC incorporation influence different aspects of cement performance.

The biological response revealed a more complex relationship. Although all formulations demonstrated cytocompatibility and osteoconductive properties, Cem4 exhibited the highest BV/TV value at 6 weeks. The 6-week time point was selected by us as optimal for comparing the effect of the cement material composition on the process of new bone tissue formation and for comparison with the control group (a defect without cement material. The increase in BV/TV associated with CMC incorporation was greater in the 60 mol.% Mg formulations than in those containing 20 mol.% Mg, suggesting a possible interaction between these factors in early bone regeneration.

The observed increase in BV/TV and bone mineral density, together with the improved functional properties and favorable histological findings, suggests that combining higher magnesium content with CMC incorporation may provide a promising strategy for optimizing calcium–magnesium phosphate bone cements. Nevertheless, advancing the investigated calcium–magnesium bone cement formulations toward clinical translation will require further study of several critical aspects—such as long-term mechanical stability, degradation kinetics under physiological conditions, and local inflammatory responses, as well as storage stability and the reproducibility of the manufacturing process—all of which will be the subject of future research.

## 4. Materials and Methods

### 4.1. Chemicals

High-purity-grade calcium nitrate tetrahydrate (≥99.0), magnesium nitrate hexahydrate (≥99.0), diammonium phosphate (≥99.0), and sodium acid phosphate (≥99.0), were acquired from Labtech (Moscow, Russia), and sodium CMC Mw~90000 Da (≥99.0) (Sigma-Aldrich, St. Louis, MO, USA).

### 4.2. Preparation of Cement Powders, Cement Liquid, and Samples

We synthesized cement powders using our developed technology, described in detail [72]. Briefly, we applied the method of precipitation from an aqueous solution according to Equation (4), followed by heat treatment at 1150 C for 60 mol.% Mg and 1350 °C for 20 mol.% Mg (air atmosphere, the dwell time (3 h), and the heating rate (10 °C/min). After sintering. the powders were ground in a vibratory mill for 60 min in air. The powders with a (Ca + Mg)/P molar ratio of 1.67 and ion substitution of Mg/(Ca + Mg) = 20 mol.% and Mg/(Ca + Mg) = 60 mol.% were obtained, with expected formation of magnesium-substituted whitlockite, stanfieldite and MgO phases.

(10-10x)Ca(NO_3_)_2_ + 10xMg(NO_3_)_2_ + 8NH_4_OH + 6(NH_4_)_2_HPO_4_ → Ca_(10-10x)_Mg10_x_(PO_4_)_6_(OH)_2_ + 20NH_4_NO_3_ + 6H_2_O, (4)

where x = 0.02; 0.06.

A solution based on 50 wt.% NaH2PO4 (called A, with pH value 3.4 ± 0.1 and characterized by viscosity 7.5 ± 0.5 mPa/s and surface tension 81.2 ± 0.3 mN/m) served as cement liquid. To improve the injectability of cement materials, sodium CMCwas introduced into the cement liquid in amounts of 0.2–1.00 wt.%. CMPC materials were prepared by mixing a cement powder with the cement liquid at a PLR of 2 g/mL under sterile conditions on a glass slide using a spatula for 1 min at ambient temperature (22 ± 2 °C. The PLR of 2 was judiciously selected based on our previous studies, based on the balance of injectability with the cement paste’s workability, considering such factors as anti-washout resistance, setting time, and mechanical strength. Cement materials with the following names were obtained: Cem1 (20%Mg+A), Cem2(60%Mg+A), Cem3(20%Mg+A+CMC) and Cem4(60%Mg+A+CMC), respectively. For following investigations three specimen geometries were used: cylinders 6 × 12 mm for mechanical testing, disks 6 × 2 mm for degradation testing, and 1 mL syringe samples for injectability testing.

### 4.3. Characterization of the Materials

Chemical composition of the powders was analyzed by inductively coupled plasma atomic emission spectrometry (AES-ICP, Agilent 720, Vista Pro, ICP Expert 4.0 Software). The uncertainty of the ICP results did not exceed 2.5%.

Phase identification was performed by X-ray diffraction (XRD) on a Shimadzu XRD-6000 diffractometer (Kyoto, Japan) employing CuKα radiation. Diffraction patterns were recorded over a 2θ range of 10° to 60° with a step size of 0.02°. Data interpretation was carried out using the ICDD PDF2 database, with phase matching against the following reference patterns: whitlockite phase (Ca_2_._589_Mg_0_._411_(PO_4_)_2_: ICDD 000-87-1582) stanfieldite (Mg_3_Ca_3_(PO_4_)_4_: ICDD 000-73-1182), newberyite (MgHPO_4_·3H_2_O: ICDD 969-00-7633), magnesium oxide (MgO: ICDD 000-77-2364), brushite (CaHPO_4_·2H_2_O: ICDD 000-11-0293), magnesium phosphate (Mg_3_(PO_4_)_2_: ICDD 000-071-1164), whitlockite (Ca_3_(PO_4_)_2_: ICDD 000-09-0169), and bobierrite (Mg_3_(PO_4_)_2_·8H_2_O: ICDD 000-16-0330). Additionally, magnesium-substituted and (Ca_18_Mg_2_H_2_(PO_4_)_14_: ICDD 000-70-2064) were taken into consideration for a more accurate evaluation of whitlockite-type structures. Quantitative phase analysis was conducted using PHAN% v6.0.11 software, with an estimated uncertainty of 5%.

### 4.4. Particle Size and Microstructural Characterization

The morphology of both the initial cement powders and the microstructure of the set cement materials were investigated by scanning electron microscopy (SEM, Tescan Vega II, Brno, Czech Republic). Elemental distribution was assessed via energy-dispersive X-ray spectroscopy (EDS) in mapping mode, utilizing an Oxford Instruments detector (Abingdon, UK). Quantitative analysis of the SEM micrographs was carried out using ImageJ software (version 1.52u), applying the secant method to a minimum of 50 line segments per sample to ensure statistical relevance.

### 4.5. Spectroscopic Analysis

Fourier-transform infrared (FTIR) spectra were recorded using a Spectrum 3 FTIR spectrometer (Perkin Elmer, Waltham, MA, USA) in attenuated total reflectance mode over the range of 400–4000 cm^−1^ with a Universal ATR accessory (diamond/KRS-5 crystal).

### 4.6. Rheological Assay

The setting time of the cementitious materials was determined in accordance with ISO 9917 (2007) using a Vicat needle apparatus equipped with a 1.0 mm diameter indenter and a total load of 400 g. The setting time was recorded as the point at which the needle no longer left a complete circular depression on the sample surface.

The pH of the cement extracts was measured in distilled water (Testo).

The viscosity of the cement liquid formulations was evaluated using a Brookfield DV2T viscometer (Brookfield Engineering Laboratories, Inc., Middleboro, MA, USA, MSA Altair) at rotational speeds of 25 and 100 rpm.

### 4.7. Injectability and Washout Resistance

Injectability was assessed by manually pushing paste-like cements through a 2 mL syringe with an inner diameter of 10 mm and wall thickness of 0.9 mm and an extrusion aperture inner diameter of 3 mm no additional needle or cannula was attached, so the extrusion aperture corresponded to the syringe outlet. The filled syringes were fixed perpendicularly in a customized matrix and positioned between the knock plates of a compression machine. The injectability of cement samples as a function of the PLR was investigated by measuring the mass of a material extruded through a syringe at constant pressure according to [130]. This method was optimized, and the analyses were performed on an Instron 5581 instrument (Norwood, MA, USA).

The methodology of the experiment consisted of several stages. At the first stage, the mixing of a cement powder with the cement liquid was performed on a glass slide with a spatula. The mixtures were blended manually for 1 min, then the cement paste was transferred into a syringe and testing was started less than 1 min after the end of mixing for all samples to ensure a comparable degree of paste maturation. The volume of the sample was 1 mL. At the second stage, the syringe with the cement paste was immersed in the die of the unit, and pressure was applied during a constant transverse movement of 1 mm/s to the punch of the syringe with a maximum distance traveled of 15 mm (velocity measurement error: 0.2%, load measurement error: 0.5%); for all samples, this piston rate corresponded to a volumetric extrusion rate of approximately 4.7 mL/min.

Injectability was calculated using the following equation:

Injectability (%) = (W1 − W2)/W1 × 100%,(5)

where W1 is the initial mass of 1 mL of the material in the syringe before the experiment, and W2 is the mass of the non-extruded material in the syringe.

The measured injectability depends on the testing parameters and should therefore be interpreted with caution. The resistance to flow is strongly governed by the extrusion aperture diameter (approximately proportional to 1/r^4^ according to the Hagen–Poiseuille relation), so the absolute values obtained here with a 3 mm aperture are not directly comparable with studies employing narrower needles or cannulas. The piston rate of 1 mm/s defines the shear rate during extrusion; higher rates may promote filter-pressing (liquid–solid separation) and reduce the apparent injectability, whereas lower rates may allow partial setting of the paste. The interval between mixing and testing is also critical, because the viscosity of the cement paste increases with time; a fixed interval was therefore used for all samples. Finally, the 1 mL sample volume and the syringe geometry determine the friction between the paste and the syringe wall and thus the force recorded by the machine.

Washout resistance and cohesion were evaluated by visual inspection via a modified method. For the anti-washout ability test, the prepared pastes were loaded into a 1 mL syringe and then directly injected into PBS solution. We observed whether there was visible disintegration in the solution before and after kept it into Ringer’s solution at 37º C for 10, 30, and 60 min by gently shaking. Disintegration of the composites was examined for [131].

### 4.8. Mechanical Testing

Cement pastes were cast into silicone cylindrical molds with an inner diameter of 6 mm and a height of 12 mm. The molds were filled with a spatula. The samples were allowed to set at 22 °C and 100% relative humidity for 24 h, then demolded and further hardened at 22 °C and 100% relative humidity for an additional 48 h (72 h in total from the time of mixing). After hardening, the specimens (*n* = 5 per group) were tested in compression. An Instron 5581) uniaxial testing machine was employed to measure the compressive strength at a crosshead speed of 1 mm/min. ASTM D695-91 was selected because the specimen dimensions (6 × 12 mm) and the brittle nature of the set cement are compatible with this standard, and the h/d ratio of 2 is consistent with the geometry recommended therein. The results are reported as mean ± standard deviation (SD).

### 4.9. Dissolution Assays

Passive-degradation in vitro assays were performed by means of weight loss, similarly to a previous article [132]. To understand degradation behavior and solubility of the CMPC samples in the form of disks (6 mm in diameter and 2 mm in height) were soaked in SBF at 37.0 °C and a *w*/*v* ratio of 0.2 g/mL and a surface area-to-volume ratio of 0.1 cm^−1^ for various periods in a closed system. Two samples were taken from the SBF at each predetermined time point (1, 7, 14, 21 and 28 days) and were then washed gently with distilled water and lyophilized dried at 60 °C for 24 h. Surface morphology and composition were determined by SEM and XRD. The ion release into the solution (i.e., into SBF) and the concentration of Ca, Mg, and Na ions in the solutions after the soaking for different periods were determined via ICP-MS measurements.

### 4.10. In Vitro Assay

Prior to in vitro studies, CMPC samples were sterilized by γ-irradiation at a dose of 18 kGy.

#### 4.10.1. Marking and Composition of the CMPC Samples

For the in vitro stage of biomedical studies of bone cements, a batch of cement powders based on the phase of magnesium-substituted vitlocite, characterized by a ratio (Ca+Mg)/P = 1.67 and calcium substitution for magnesium 20 mol. % and 60 mol%. For cementing, cementing liquids (CL) based on sodium acid phosphate (samples Cem1 and Cem2) and 0.75% CMC (samples Cem3 and Cem4) were used. The cemented samples were ground and sieved on a series of sieves. The 300–600 µm fraction was used in the experiments.

#### 4.10.2. Cell Lines

The test culture used in the work was the human osteosarcoma cell line MG-63 (Russian Collection of Cell Cultures, Institute of Cytology, Russian Academy of Sciences, St. Petersburg, Russia), which was maintained at an initial seeding density of 2.0 × 10^4^ cells/cm^2^ in a complete growth medium (CGM) of the following composition: DMEM medium (PanEco, Moscw, Russia), 10% fetal bovine serum (FBS, HyClone, Logan, UT, USA), 0.58 mg/mL L-glutamine and 20.0 µM Hepes solution (PanEco, Moscw, Russia), 1% (*v*/*v*) penicillin/streptomycin solution (PanEco, Moscow, Russia).

Experiments to assess the osteodifferentiation potential of CMPC samples were performed on a culture of (donor bone marrow mesenchymal stromal cells BM hMSCs) (informed consent of the donor was approved at a meeting of the Local Ethics Committee of the Moscow P.A. Hertzen Research Oncological Institute; protocol No. 637 dated 15 February 2021) at the 4th passage, which was maintained at a seeding density of 5.0 × 10^3^ cells/cm^2^ in CGM of a similar (as for MG-63) composition, with the exception of the culture medium-DMEM/F12 (PanEco, Moscow, Russia).

In order to check the “stemness” of the donor BM hMSC culture, immunophenotyping of this culture was performed using a special set of monoclonal antibodies (MABs) to clusters of differentiation: CD105, CD90, CD73, CD45, CD34 (Miltenyi Biotec, Gladbach, Germany), flow cytofluorometer Cytoflex (Beckman Coulter Inc., Miami, FL, USA). In addition, the ability to induced differentiation in the osteogenic direction was determined by culturing BM hMSCs in a special differentiation medium (StemPro Osteogenesis Differentiation Kit, Vancouver, BC, Canada). Upon completion of the period of directed osteogenic differentiation, a marker of osteogenic differentiation, alkaline phosphatase, was determined in the cells (alkaline phosphatase staining kit, Himedia, Mumbai, India). BM hMSCs with the phenotype CD90+, CD105+, CD73+, CD34−, and CD45−, capable of osteogenic differentiation, were used in the work.

MG-63 cells and BM-hMSCs were passaged using a standard method, with medium changes twice weekly. Once the culture reached 80% confluency, the cells were suspended using a 1:1 mixture of 0.25% Trypsin and Versen (PanEco, Moscow, Russia), counted on a Countess II FL automated cell counter (Invitrogen, Thermo Fisher Scientific, Carlsbad, CA, USA), and then seeded into culture flasks (Corning Inc., Corning, NY, USA) at a 1:3 ratio.

All manipulations with materials and cells were performed in a sterile environment in a Class II laminar flow hood (Heraeus, HERAsafe, Hanau, Germany). Cell plates were cultured in a humidified CO_2_ incubator (Sanyo MCO-20AIC, Sanyo Electric Co., Osaca, Japan) at 37 °C and 5% CO_2_.

#### 4.10.3. Cell Viability Methods

(a) MTT Assay for Assessing Cell Metabolic Activity

This method is based on the ability of living cell dehydrogenases to reduce 3-(-4,5-dimethylthiazolyl-2)-2,5-diphenyltetrazolium bromide (MTT, Sigma Aldrich solutions, Merck SA, Darmstadt, Germany) into blue, water-insoluble formazan crystals [133]. To perform the MTT assay, at the end of cell growth period half of the growth medium (100 µL) was removed from each well, and 25 µL of MTT solution at a concentration of 5 mg/mL was added to each well if the experiment was conducted in 96-well plates. When using 48-well plates, 200 µL from 400 µL was removed and 50 µL of MTT was added to each well. After 3 h of incubation (5% CO_2_, 37 °C), the entire volume of medium was decanted from each well. The resulting formazan was dissolved using isopropyl alcohol (Khimmed, Moscow, Russia) at a volume of 200 µL per well in a 96-well plate or 400 µL per well in a 48-well plate, accordingly. The precipitate formed as a result of protein precipitation in isopropanol was removed by centrifuging the plates for 10 min at 3000 rpm (Jouan, Saint-Herblain, France). Next, 100 µL of supernatant from each well was transferred to a 96-well flat-bottomed plate (EIA/RIA, Corning Inc., Corning, NY, USA), and the optical density (OD) of the formazan solution was assessed using a Multiscan FC spectrophotometer (Thermoscientific, Life Technologies, Carlsbad, CA, USA) at a wavelength of 540 nm.

For each test sample at a specific time of cell growth, the population of the viable cells (PVC) was calculated in the experiment relative to the control (cells on polystyrene, in %) using the formula:

PVC = ODexp.: ODcontr. × 100%,(6)

where OD is the optical density of the formazan solution in the experiment and negative control, respectively. Next, when assessing the toxicity of the samples, according to GOST R ISO 10993.5-99, the toxicity index (TI) was calculated using the formula:

TI = 100% − ODexp.:ODcontr. × 100%(7)

A sample was considered non-toxic if the PVC value at the experimental stages was ≤30%, and cytocompatible if the PVC value at the experimental stages was ≥70%.

At the stages of the experiment, visual monitoring of the processes of cell expansion on the surface of the cement samples and photo archiving of materials (Olympus stereomicroscope system SZX7, Olympus, Tokyo, Japan) were performed.

(b) Live/Dead Test

Cell viability was determined using the Live/Dead kit (Molecular Probes, Invitrogen ThermoFisher Scientific, Life Technologies, Carlsbad, CA, USA) according to the manufacturer’s protocol for visualizing live and dead cells by undirect contact of the MG-63 culture (cell growth in extracts of the studied bone cement samples). Simultaneous fluorescent staining of live (using calcein-acetoxymethyl (Calcein-AM), green fluorescence at λ = 517 nm) and dead (using ethidium homodimer (Eth-1), red fluorescence at λ = 617 nm) cells was performed using a Nikon Eclipse TI fluorescence microscope (Tokyo, Japan).

The toxicity of the bone cement samples was determined according to ISO 10993 (parts 5 and 12) [89]. using the indirect contact method by MG-63 cell growth in extracts of the bone cement samples. Extracts of the hardened cements were prepared under sterile conditions in a thermostat at 37 °C for 24 h on a shaker (ELMI, Riga, Latvia) at 170 rpm. CGM was used as the extraction fluid. In accordance with ISO 10993-12-2015, the mass of material to volume of extraction fluid ratio during extraction was 0.2 g/mL ± 10% [88].

Particles of the cement’s material that became trapped in the extracts during their preparation were removed by centrifugation (3000 rpm, 15 min, Eppendorf Centrifuge 5810 R, Hamburg, Germany). The extracts were used immediately after preparation.

The seeding density of human osteosarcoma MG-63 cells was 2.0 × 104 cells/cm^2^ (96-well culture plates, Corning Inc., Corning, NY, USA) in a volume of 200 μL of CGM. The extracts of the samples were added to the cells 24 h after seeding into the wells of a subconfluent MG-63 culture monolayer. For this purpose, the culture medium was completely decanted from the wells, and the extracts were added at a volume of 200 μL per well. Pure CGM was added to the cells as a negative control, and a 50% solution of dimethyl sulfoxide (DMSO, PanEco, Moscow, Russia) in CGM served as a positive control. At least five replicates were used for extracts from each material sample and control. The cells were grown in the presence of bone cement extracts for 24 and 72 h. Then, the cell viability was determined using the MTT- and Live/Dead assays and the PVC value and TI were calculated.

#### 4.10.4. Investigation of Adhesion Properties of the Surface of CMPC Samples

To evaluate the degree of cell adhesion to the surface of bone cement samples, the cell area was determined after 24 and 72 h of cells growth. For this purpose, cement samples were placed at the bottom of 8-well slide culture bottles (Corning Inc., Corning, NY, USA), with each containing 1 type for each incubation period. Then, a suspension of MG-63 cells at a concentration of 1.0 × 10^4^ cells/cm2 was added to the wells with (experiment) and without samples (control, polystyrene). MG-63 cells grew for 24 and 72 h.

At the indicated times, the cells were fixed with a 4% paraformaldehyde solution (BioVitrum, St. Petersburg, Russia) for 10 min at room temperature and washed three times with phosphate-buffered saline (PBS, PanEco, Moscow, Russia). Then, they were treated with a 0.05% Triton-X100 solution (PanEco, Moscow, Russia) for 10 min at room temperature, followed by washing three times in PBS.

To block nonspecific antibody binding, cells were treated with a 10% solution of secondary antibody donor serum (goat serum) for 30 min at room temperature (Krollinfo, Moscow region, village of Novaya, Russia). Immediately after the blocking step, cells were incubated with primary human monoclonal antibodies to vinculin (Sigma Aldrich solutions, Merck SA, Darmstadt, Germany) at a dilution of 1:200 in a humid chamber, which was carried out overnight at +4 °C.

After washing the cells from the primary antibody solution in PBS, the Alexa Fluor 488 Goat anti-mouse secondary antibody (Thermo Fisher Scientific, Life Technologies, Carlsbad, CA, USA) was used at a dilution of 1:1000. The cells were incubated for 1.5 h at room temperature in the dark.

Rhodamine-Falloidin (Thermo Fisher Scientific, Life Technologies, Carlsbad, CA, USA) at a working dilution of 1:1000 was used to stain the cytoskeleton of cells when cultured on samples; it was incubated for 1.5 h at room temperature in the dark, according to the manufacturer’s recommendations. After cells were washed with PBS to remove unbound dye, nuclei were stained with DAPI (Thermo Fisher Scientific, Life Technologies, Carlsbad, CA, USA).

Images of MG-63 human osteosarcoma cells, labeled with primary antibodies against vinculin and secondary antibodies conjugated with Alexa Fluor 488 and DAPI, were acquired on an A1R confocal system mounted on a Nikon Ti microscope (Nikon, Tokyo, Japan).

Aqueous immersion and a Nikon CFI Plan Fluor 20x MImm DIC N2× 0.75 NA objective (Nikon, Tokyo, Japan) were used for imaging. Excitation/emission wavelengths were ex = 405 nm/em = 450 (50) nm (DAPI) and ex = 488 nm/em = 525 (50) nm (FITC, Alexa Fluor 488). Images were processed and counted using NIS Elements ver.4.51 software (Nikon, Tokyo, Japan).

Five fields of view containing an average of 200 cells after 24 h and 72 h of cell growth were counted for each.

#### 4.10.5. The Influence of CMPC Extracts on the Migration Capacity of MG-63 Cells

To evaluate the effect of bone cements on the migration capacity of MG-63 cells, a scratch test was used [134]. For this, the cells were seeded in 96-well plates in triplets for each sample (Corning Inc., Corning, NY, USA) at a density of 8.0 × 10^3^ cells per well in 200 μL of CGM. Upon reaching a confluent monolayer, the growth medium was decanted from the wells and a standard-sized monolayer defect was created using a special scraper. Next, 200 μL volumes of Cem1, Cem2, Cem3, and Cem4 extracts were added to the wells at dilutions of 25%, 50%, 75%, and 100%. As a control, the cells were cultured in CGM. Next, the plates were placed in a YuLI Stage intravital cell monitoring device (Nano Entek, Seoul, Korea), and the closure of the MG-63 monolayer defect was observed according to the manufacturer’s Scratch Basic protocol. Filming was performed every three hours for 25 cycles. Three independent experiments were carried out. The results were processed using the YuLI Stat program.

#### 4.10.6. In Vitro Cytocompatibility Assessment of CMPC Samples (Direct Contact Method)

In vitro cytocompatibility was performed by direct contact of the test culture with the surface of the test samples. Sterile bone cement samples were placed in wells of 48-well culture plates (SPL, Gyenggi-do, Korea) (four samples of each type for each experimental period: three for cytocompatibility assessment and one for an optical blank). The cell suspension was added at a seeding density of 1.0 × 10^4^ cells/cm2 in a volume of 1 mL. Cultivation was performed for 1, 3, 7, and 10 days with regular (twice weekly) medium changes. Wells containing cells on polystyrene culture plastic served as controls. At the indicated culturing times, MG-63 cells viability was determined and the PVC value was calculated.

#### 4.10.7. Study of the Ability of CMPC Samples to Support Osteogenic Differentiationof Donor BM-MSCs In Vitro

To assess the osteogenic plasticity of BM-hMSCs when cultured on non-toxic experimental bone cement samples, the cells were cultured in CGM in 6-well plates (Corning Inc., Corning, NY, USA) at an initial seeding density of 5.0 × 10^3^ cells/cm2. When the culture reached a preconfluent monolayer (days 7–9 of the experiment), the CGM in the wells was replaced with a special osteogenic medium (OM) (Differentiation Basal Medium and MesencultTM Differentiation 5× Supplement, Stemcell Technologies, Vancouver, BC, Canada). Osteogenic differentiation gene expression was determined using real-time polymerase chain reaction (RT-PCR) on day 21 after differentiation was initiated.

According to the experimental design, the following groups were formed:

1. Donor BM-hMSCs were seeded on plastic in CGM (control 1);

2. Donor BM-hMSCs were seeded on plastic in a special OM (control 2);

3–6. Donor BM-hMSCs were seeded on Cem1-Cem4 experimental samples accordingly, in the presence of CGM;

7–10. Donor BM-hMSCs were seeded on Cem1-Cem4 experimental samples, accordingly in a special OM.

#### 4.10.8. Determination of the Expression of Several Osteodifferentiation Marker Genes (Real-Time Polymerase Chain Reaction (RT-PCR))

The changes in the expression level of osteogenic differentiation marker genes in cells were assessed using real-time PCR. On the 21st day of cell growth in preconfluent conditions, the following genes were analyzed: RUNX2, ALPL, and SP7. The expression of the abovementioned genes was normalized using theglyceraldehyde-3-phosphate dehydrogenase gene (“housekeeping” gene, GAPDH). The total RNA pool was isolated by phenol-chloroform extraction using Qiazol lysis buffer (Qiagen, Hilden, Germany) followed by purification on RNeasy Mini Kit columns (Qiagen, Hilden, Germany). The amount of RNA in the samples was measured using a NanoDrop ND-2000 spectrophotometer (Thermo Scientific, Waltham, MA, USA). Reverse transcription was performed using the MMLV RT kit (Evrogen, Russia) using Random (dN)10-primer. qPCRmix-HS SYBR with SYBR Green I intercalating dye (Evrogen, Moscow, Russia) and a DTlite detecting amplifier (DNA-Technology, Moscow, Russia) were used for real-time PCR assay. The amplification program included a “hot start” (94 °C, 10 min) followed by 50 cycles of template denaturation (94 °C, 20 s), primer annealing (64 °C, 10 s), and amplicon elongation (72 °C, 15 s). All samples were analyzed in triplets for three independent experiments. The gene of interest expression was normalized to GAPDH [135] using the ΔΔCt method. As a result, the fold-change (FC) in the expression levels of the studied genes in the test and control groups were calculated. The control was non-treated MSCs at day 0, incubated under standard conditions (CGM). Synthesis of oligonucleotide sequences of primers of target and normalization gene was carried out by Evrogen (Moscow, Russia). Sequences of primers for marker genes were designed using the NCBI Primer-BLAST tool (www.ncbi.nlm.nih.gov/tools/primer-blast accessed on 5 March 2026); the sequences are shown in Table 4.

#### 4.10.9. Alkaline Phosphatase Determination During Differentiation of Donor BM-MSCs into Osteoblasts

ALP activity (qualitative analysis) in the cytoplasm of BM MSCs was determined using the EZstain Alkaline Phosphatase Staining kit (CellCulture, HIMEDIA, Mumbai, India) according to the manufacturer’s protocol. For the test, fixed preparations (citrate-acetone-formaldehyde fixative—30 s at room temperature) were incubated at room temperature for 30 min in a solution containing naphthol AS-BI phosphate and a freshly prepared diazonium salt solution (the latter was prepared by mixing sodium nitrite solution with FRV red-violet solution, adjusted to pH 9.5 with 2-amino-2-methyl-1,3-propanediol). The resulting red-violet color indicates sites of ALP activity.

### 4.11. In Vivo Assays

#### 4.11.1. Laboratory Animals and Models

The biocompatibility of Cem1–Cem4 samples was studied using a model of their subcutaneous implantation., osteoconductive potencies-in a bone defect model (marginal resection of the rat tibia). Animals were obtained from Federal State Budgetary Institution of Science “Scientific Center for Biomedical Technologies of the Federal Medical and Biological Agency of Russia” (Andreevka branch, Moscow Region, Russia). In the in vivo experiments, a simple randomization method was used: inbred animals (Wistar rats, B/D mice) of the same sex (males) and similar body weight (18–20 g for mice; 180–220 g for rats) were employed. The animals were randomly assigned to experimental groups. To ensure independent results, a blinding procedure was applied. The bone substitute material samples (CMPC) were coded.

Exclusion criteria for the animals included the development of postoperative infections, fractures of the limb in the area of the defect, or instances where an animal caused self-inflicted injury to that area.

Experiments on laboratory animals were carried out in compliance with the principles of humanity and the requirements set out in the Council Directive 2010/63/EU “On the use of animals for experimental research”, as well as in accordance with the developed protocol for in vivo experiments to study the biocompatibility and osteoplastic potential of the samples of the bone cements, which was approved by the Commission for Bioethical Control over the Care and Use of Laboratory Animals for Scientific Purposes of the Federal State Budgetary Institution “Scientific Medical Research Center of Radiology” of the Ministry of Health of the Russian Federation (protocol No. 2-N-00060 dated 28 March 2024).

#### 4.11.2. Studies of Biocompatibility of the CMPC Samples

To evaluate biocompatibility, a batch of Cem1–Cem4 samples was prepared in the form of tablets 5 mm in diameter and 2 mm in height. Prior to surgery, the cement samples were sterilized with γ-irradiation at a dose of 18 kGy.

Mice were surgically anesthetized under general anesthesia (Vesotil 100 (VetStem, Moscow, Russia)–35 mg/kg and Xylanite (Nita-Farm, Saratov, Russia)–7.5 mg/kg, intraperitoneally). A transverse skin incision was made on the animals’ backs near the shoulder blades. The skin was carefully separated from the adjacent subcutaneous tissue and muscle using dissecting tweezers. A saline-soaked bone cement samples were implanted into the resulting “pocket.” The wound was sutured, after which the animals were observed.

At designated postoperative times (2, 4, 8, and 12 weeks), mice were euthanized (CO_2_ chamber, Technoplast, Buguggiate, Italy), three animals at each time point. Skin samples were removed and visually assessed using a stereomicroscope and digital video camera (Olympus, Japan). They were then fixed in 10% buffered formalin and decalcified in Medix solution (Medix LLC, Moscow, Russia) for 14 days, with daily replacement of the decalcifying solution. Paraffin blocks were prepared. Histological preparations were stained with hematoxylin and eosin.

#### 4.11.3. Studies of Osteoconductivity Potencies of the CMPC Samples

To evaluate the osteoplastic properties of the developed bone cements, the entire cortical bone defect in rats was restored with freshly mixed bone cements samples according to the experimental protocol. Prior to surgery, the cement powders were sterilized at 160 °C (Binder dry-heat oven, Tuttlingen, Germany), and the cement liquid was filtered (Millipore filters, 0.22 μm pore diameter, Merck, Darmstadt, Germany).

**Figure 18:**
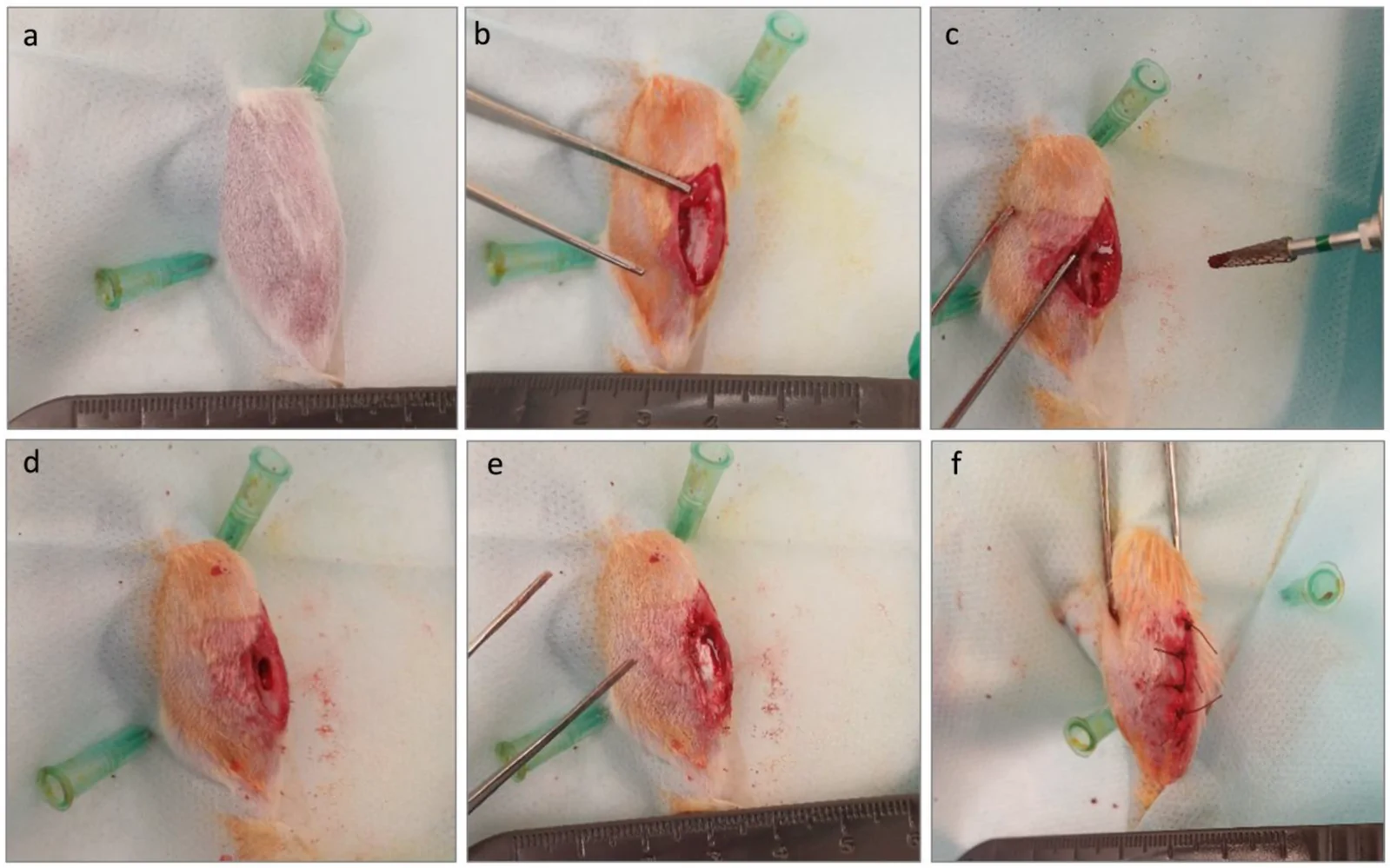
Stages of formation of monocortical defect of the rat’s tibia: (**a**) anterior surface of the tibia; (**b**) exposure of the outer surface of the tibia; (**c**,**d**) creation of a bone defect using a dental bur; (**e**) filling of a bone defect with freshly prepared CMPC; (**f**) layer-by-layer closure of the completed bone defect.

To create a monocortical defect, animals underwent surgery under general non-inhalation anesthesia: Vesotil 100 (VetStem, Russia) at a dose of 35 mg/kg and Xylanite (NITA-FARM, Russia) at a dose of 7.5 mg/kg. The drugs were administered intramuscularly, once, sequentially. After preparing the surgical field (shaving the thigh area with a Moser clipper, Baden-Württemberg, Germany) and subsequent treatment with the antiseptic solution Abacteril Active (OOO Rudez, Moscow, Russia), a skin incision approximately 2 cm long was made along the anterior surface of the middle third of the tibia in rats (Figure 18a). The muscles were then bluntly retracted, exposing the outer surface of the tibia for approximately 1.0–1.5 cm (Figure 18b). Using a dental bur (Figure 18c), a “fenestrated” bone defect was created in the animals: two holes were made in the outer cortical layer, spaced 5–7 mm apart, this defect was then sawed within the designated boundaries, and the contents of the medullary canal were removed with a spatula (Figure 18d). The resulting bone defect was filled with freshly prepared bone cement according to the experimental protocol (Figure 18e), covered with adjacent tissue, and the wound was sutured (Figure 18f).

Postoperative care of the animals consisted of antibacterial and anti-inflammatory therapy (4% Tolfedine solution (Vetoquinol, Gorzów Wlkp, Poland) at a dose of 4.0 mg/kg, intramuscularly, once) daily during the first week.

At 3, 6, and 12 weeks and 6 months, three animals from each group were sacrificed by euthanasia using carbon dioxide. (CO_2_ chamber, Tekhnoplast, Bavaria, Germany). The tibia sections containing the defect were removed, fixed in 10% neutral formalin, and decalcified with Medics solution (Medix LLC, Russia) for 30–40 days. The biological material samples were then dehydrated in graded alcohols, embedded in paraffin, histological preparations were made and stained with hematoxylin and eosin.

Light microscopy of the histological preparations was performed using an Eclipse Ti microscope equipped with a DS-Fi1c digital camera (Nikon, Japan).

Experimental animals (48 mice and 60 rats) were divided into four experimental groups of three animals each, plus a control group (rats):

Group 1—implantation of Cem1 sample;

Group 2—implantation of Cem2 sample;

Group 3—implantation of Cem3 sample;

Group 4—implantation of Cem4 sample;

Group 5 (rats, bone defect under a blood clot, control).

#### 4.11.4. Micro-CT of the CMPC Implantation Area

To evaluate the process of new bone formation in rats after the defect site was replaced with bone cement samples over time, sections of the tibia with the defect were excised after 3, 6, and 12 weeks of the experiment. A SkyScan 1275 laboratory micro-CT system (Bruker, Kontich, Belgium) was used for the study. An accelerating voltage of 20 kV and a beam current of 175 µA were selected. The effective pixel size was set to 4.5 µm. A total of 1200 angular projections were acquired over a 360° range. Nine images were acquired at each angular position with an exposure time of 0.6 s. After reconstruction using the manufacturer’s software (NRecon version NRecon V2, Bruker), the resulting dataset was imported into VGStudio MAX 2.1 software for visualization and segmentation of structures of interest.

A volume of interest (VOI) corresponding to the defect area was selected for analysis. Bone tissue was segmented using a global threshold in gray units, determined from the density distribution histogram. The binarization threshold was selected visually by comparing the segmented image with the original slice for each sample and was maintained constant within each time point. The bone volume fraction was calculated as the ratio of bone volume (BV) to the total volume of interest (TV), expressed as BV/TV.

The mean VOI density was calculated as:

ρ_VOI_ = (BV/TV)× ρ_bone_ + (1-BV/TV)×ρ_marrow_ (8)

where ρ_bone_ and ρ_marrow_ are the densities of mineralized bone and bone marrow, respectively. As no hydroxyapatite phantom calibration was performed, literature values were used: ρ_bone_ = 1000 mg HA/cm^3^ and ρ_marrow_ = 50 mg HA/cm^3^ [136,137]. The resulting density values should therefore be considered semi-quantitative estimates rather than absolute measurements.

A conservative approach was used to assess uncertainty: both random (noise, segmentation) and systematic (calibration, beam hardening) sources were taken into account. The final relative uncertainty of ±6% represents the upper bound of the combined uncertainty and provides a margin for sample comparison. Data are presented as mean ± SD (*n* = 3). Statistical analysis was performed using Student’s t-test; significance was set at *p* < 0.05.

The relative increase in bone tissue was calculated as:

(9) $$\mathrm{Relative}\;\mathrm{increase}\;(\%)\;=\frac{BV/TVfinal-BV/TVinitial}{BV/TVinitial}\times 100\%$$

### 4.12. Statistical Analysis of Results

The results were processed using standard methods of variation statistics using Microsoft Excel 2000. The significance of differences was assessed using the parametric Student’s t-test. Differences were considered statistically significant at *p* < 0.05.

## 5. Conclusions

Thus, based on the comprehensive study conducted, it can be concluded that the developed bioactive injectable CMPCs, prepared using powders containing 20 and 60 mol% Mg^2+^ and additional CMC solution introduced in a sodium phosphate solution as the cement liquid, exhibited improved handling and functional properties, including appropriate setting time, mechanical strength, and a favorable bioresorption rate. The introduction of CMC significant improved injectability of the cements, especially for high magnesium content composition (Cem4). Moreover, the developed CMPCs demonstrated promising biological performance both in vitro and in vivo, including supporting osteogenic differentiation of BM hMSCs. The increased Mg^2+^ concentration in Cem4 composition promoted acceleration of new bone tissue formation and could therefore be considered a promising osteoinductive biomaterial, following extensive and multifaceted preclinical testing. Taken together, these properties support the potential for further clinical application of the developed CMPCs in reconstructive surgery, orthopedics, and maxillofacial surgery.

## Figures and Tables

**Table 1:** Viscosity and surface tension of cement liquids.

Cement Liquid	Concentration of CMC, %	Viscosity, mPa s		Surface Tension, mN/m	pH Level
		50 rmp	200 rpm		
A	0	7.5 ± 0.5	20.2 ± 0.5	81.2	3.4 ± 0.1
A + CMC	0.2	9.0 ± 0.5	25.8 ± 0.5	73.1	3.8 ± 0.1
	0.5	33.0 ± 0.5	56.5 ± 0.5	73.1	3.9 ± 0.1
	0.75	91.5 ± 0.5	52.6 ± 0.5	76.4	3.9 ± 0.1
	1.0	235.5 ± 0.5	15.7 ± 0.5	77.9	4.0 ± 0.1

**Table 2:** Contact angles for cement liquids.

Surface Type	Cement Liquid	CMC Concentration, %	Left	Right
Teflon	A	0	119.6	116.6
	A + CMC	0.2	110.9	110.2
		0.5	110.2	110.3
		0.75	115.6	114.2
		1.0	112.3	112.7
Glass	A	0	54.4	51.8
	A + CMC	0.2	53.7	50.5
		0.5	52.6	57.0
		0.75	53.4	49.4
		1.0	48.1	50.5

**Table 3:** Bone morphometry (BV/TV) and mean VOI density in control group (defect without cement) and bone cement groups (Cem1–Cem4) at 6 weeks (mean ± SD, *n* = 3).

Group	BV/TV (Mean ± SD)	BV/TV (%)	ρ_VOI (mg HA/cm^3^)	Relative Increase vs. Control at 6 Weeks (%)
Control	0.179 ± 0.008	17.9	220.1	-
Cem1	0.163 ±0.010	16.3	204.9	-
Cem2	0.168 ± 0.010	16.8	209.6	-
Cem3	0.165 ± 0.010	16.5	206.8	-
Cem4	0.214 ± 0.013	21.4	253.3	+19.6

**Table 4:** Gene symbols and sequences of primers for the analyzed and normalization genes.

№	Gene Symbol	Encoded Protein	F and R Primer Sequences 5′-3′
1	*RUNX2*	Runt-associated transcription factor 2 is one of the key regulators of osteoblastic cells	*F: tca-acg-atc-tga-gat-ttg-tgg-g* *R: ggg-gag-gat-ttg-tga-aga-cgg*
2	*SP7*	Osterix is a transcription factor involved in the differentiation of mesenchymal progenitors into osteoblasts and osteocytes	*F: ccc-acc-tac-cca-tct-gac-tt* *R: gct-gcc-cac-tat-ttc-cca-ct*
3	*ALPL*	Alkaline phosphatase is a membrane-bound glycosylated enzyme involved in matrix mineralization	*F: acc-acc-acg-aga-gtg-aac-ca* *R: cgt-tgt-ctg-agt-acc-agt-ccc*
4	*GAPDH*	Glyceraldehyde-3-phosphate dehydrogenase, housekeeping gene	*F: gaa-ggt-gaa-ggt-cgg-agt-c* *R: gaa-gat-ggt-gat-ggg-att-tc*

## Data Availability

Data is contained within in article or Supplementary Materials.

## Supplementary Materials

The following supporting information can be downloaded at: https://www.mdpi.com/article/10.3390/ijms27219974/s1.
