# Supplementary material for: Injectable Bioactive Calcium–Magnesium Phosphate Cements Enable Balanced Material Resorption and New Bone Formation
Source: Int J Mol Sci. 2026 Nov 6;27(21):9974. doi: 10.3390/ijms27219974 (PMC13764586; doi:10.3390/ijms27219974)
Supplement: Supplementary file 1 [file ijms-27-09974-s001.zip › Supplementary to Figure.pdf]

Supplementary to Fig. 6, a.

The magnitude of confidence intervals and the p-value in the assessment of the cytotoxicity of Cem1 – Cem4 samples.

Samples	Confidense interval, 24 h	Confidense interval, 72 h	p-value
**Cem1**	5,19	5,29	0,000
**Cem2**	2,74	1,36	0,000
**Cem3**	3,14	5,59	0,005
**Cem4**	5,19	3,82	0,002
**DMSO**	2,06	0,10	0,000

Supplementary to Fig. 7, e, f

The magnitude of confidence intervals and the p-value in the assessment of restoration of the monolayer defect of cell lines (Scratch test).

Dilution of extracts (%)	Control (confidence interval)	Cem1 vs control		Cem2 vs control		Cem3 vs control		Cem4 vs control	
		Confidence interval	p	Confidence interval	p	Confidence interval	p	Confidence interval	p
**25%**	2.47	1.96	0.003	3.11	0.116	1.68	0.073	5.57	0.615
**50%**		2.20	0.154	1.19	0.552	4.34	0.014	2.48	0.538
**75%**		3.04	0.302	1.73	0.429	1.31	0.000	1.81	0.418
**100%**		1.96	0.503	4.13	0.002	3.92	0.002	5.81	0.014

Samples	Cem1 vs Cem2 (p)	Cem3 vs Cem4 (p)	Cem1 vs Cem3 (p)	Cem2 vs Cem4 (p)
**Cem1**				
**Cem2**	0.001			
**Cem3**			0.001	
**Cem4**		0.673		0.893

Supplementary to Fig. 9, e

The magnitude of the p-value in the assessment of the cell area of Cem1 – Cem4 samples.

Cements	p-values			
	On the CMPC samples vs control		On the glass near next to the CMPC samples vs control	
	24 h	72 h	24 h	72 h
**Cem1**	0.000	0.000	0.485	0.542
**Cem2**	0.000	0.000	0.442	0.320
**Cem3**	0.000	0.000	0.556	0.233
**Cem4**	0.000	0.000	0.874	0.253

Cell area (µm²) at different time cell growth (hours)		p-values			
		Cem1 vs Cem2	Cem3 vs Cem4	Cem1 vs Cem3	Cem2 vs Cem4
24 h	On the CMPC samples	0.715	0.321	0.056	0.373
	On the glass near next on the CMPC samples	0.881	0.709	0.836	0.569
72 h	On the CMPC samples	0.298	0.906	0.593	0.603
	On the glass near next on the CMPC samples	0.611	0.610	0.349	0.910

Supplementary to Fig. 10, a, b

The magnitude of confidence intervals and the p-value in the assessment of the cytocompatibility of Cem1 – Cem4 samples.

Incubation time (day)	Control/ Samples	Confidense interval	p-values				
			Samples vs Control	Cem1 vs Cem2	Cem3 vs Cem 4	Cem1 vs Cem3	Cem2 vs Cem4
**1**	Control	0,0124					
	Cem1	0,0124	0,006				
	Cem2	0,0231	0,003	0,040			
	Cem3	0,0141	0,006			0,774	
	Cem4	0,0067	0,003		0,596		0,027
**3**	Control	0,0328					
	Cem1	0,0101	0,000				
	Cem2	0,0192	0,000	0,013			
	Cem3	0,0554	0,001			0,101	
	Cem4	0,0407	0,000		0,717		0,967
**7**	Control	0,1379					
	Cem1	0,1388	0,339				
	Cem2	0,0668	0,001	0,000			
	Cem3	0,1629	0,046			0,137	
	Cem4	0,1926	0,866		0,088		0,003
**10**	Control	0,1409					
	Cem1	0,1431	0,118				
	Cem2	0,1944	0,696	0,141			
	Cem3	0,2668	0,207			0,864	
	Cem4	0,0672	0,022		0,697		0,041

Supplementary to Fig. 11, a

Relative gene expression levels (fold change) in the experimental group of BM-MSCs + osteogenic medium and bone cement compared to the control – untreated cells (normalized to the reference gene GAPDH).

Culture conditions	Fold Change \ $2^{-\Delta\Delta Ct}$	Standard deviation (SD)	*p*-value	Statistical significance
ALPL (alkaline phosphatase) gene expression				
MSC+OM	32.05	5.37	-	-
+Cem1	13.71	2.95	0.0375	p < 0.01 **
+Cem2	18.11	2.57	0.0418	p < 0.05 *
+Cem3	14.68	4.23	0.0082	p < 0.01**
+Cem4	26.25	3.41	0.6940	n.v.
RUNX2 (Runt-associated transcription factor 2) gene expression				
MSC+OM	3.16	0.35	-	-
+Cem1	1.76	0.19	0.0213	p < 0.05 *
+Cem2	1.84	0.41	0.0309	p < 0.05 *
+Cem3	2,24	0.38	0.0486	p < 0.05 *
+Cem4	2,79	0.27	0.7591	n.v.
SP7 (Osterix transcription factor) gene expression				
MSC+OM	11.54	1.15	-	-
+Cem1	10.84	2.11	0.3187	n.v.
+Cem2	14.76	1.98	0.0479	p < 0.05 *
+Cem3	9.48	1.87	0.1265	n.v.
+Cem4	18.25	2.54	0.0077	p < 0.01**

Note to the table: Data are presented as the geometric mean Fold Change. The control group is set to 1.0. Statistical analysis was performed between osteogenic medium only and bone cement groups using a paired Student's (*t*-test) based on Δ Δ Ct values. Significant differences of $p < 0.05$ and $p < 0.01$ are indicated as * and **, respectively; "n.s." (not significant) denotes a lack of statistically significant differences.

Supplementary

Key characteristics of the developed CMPC samples

Material property	Cem1	Cem2	Cem3	Cem4
The chemical composition	20%Mg+A	60%Mg+A	20%Mg+CMC	60%Mg+CMC
Phase powder composition	Whitlockite +MgO	stanfieldite+ MgO	whitlockite+MgO	stanfieldite+MgO
Cement liquids	A	A	CMC	CMC
Particle size of cement slurries	20 µm	50 µm	20 µm	50 µm
pH level of cement slurries	3.4	3.4	3.9-4.0	3.9-4.0
Setting temperature (5 min), °C	28	27	28	27
Setting time, min	10	13	20	23
Porosity, %	32	40	38	45
Mechanical properties, MPa	21	22	22	26
Cohesion	-	-	+	+
The mass loss (28 days), %	12	11	11	8
Injectability, %	25	5	65	50
Mg release (28 days), ppm	688	601	738	569
Cititoxicity	non-toxic	non-toxic	non-toxic	non-toxic
Cytocompatibility	+	+	+	+
Migration activity (defect closure time in the experiment/ control, 41.3h)	40.0 h	50.5 h	52.0 h	50.0 h
Effect of CMPC on BM hMSC in vitro osteodifferentiation compared to the control	ALPL < RUNX2 < SP7=	ALPL < RUNX2 < SP7 >	ALPL < RUNX2 < SP7 =	ALPL = RUNX2 = SP7 >
Biocompatibility	+	+	+	+
Osteoplastic potencies	Osteoconductivity ++	Osteoconductivity ++	Osteoconductivity ++	Osteoconductivity +++
Radiopacity	+	+	+	+
Mean VOI density in bone (6 week), mg HA/cm^3	204.9	209.6	206.8	253.3
